# A new multisystem *ERCC1*-hepatorenal syndrome: insights from a clinical cohort, molecular pathogenesis, and management guidelines

**DOI:** 10.1038/s41431-025-01910-0

**Published:** 2025-07-19

**Authors:** Susan M. White, Annelotte P. Wondergem, Isa Breet, Maren Dittmaier, Katrina Bell, Christopher M. Richmond, Winita Hardikar, Kanika Bhatia, Catherine Quinlan, David Orchard, Areetha D’Souza, Walter J. Chazin, Christopher Smith, Rebecca Sparkes, Simon Lam, Alexandra Carter, Robert J. Hopkin, Leticia Khendek, Bonnie R. Sullivan, Naja Becher, Anne Katrine W. Simonsen, Helene Kvistgaard, Katherine Dempsey, Alexander G. Miethke, Pernille Axél Gregersen, Eliza Phillips, Martijn S. Luijsterburg

**Affiliations:** 1 Victorian Clinical Genetics Services, Murdoch Children’s Research Institute, Parkville, VIC Australia; 2 Department of Paediatrics, University of Melbourne, Parkville, VIC Australia; 3 Department of Human Genetics, Leiden University Medical Center, Leiden, The Netherlands; 4 Murdoch Children’s Research Institute, Melbourne, VIC Australia; 5 Genetic Health Queensland, Royal Brisbane & Women’s Hospital, Brisbane, QLD Australia; 6 School of Medicine, Griffith University, Gold Coast, QLD Australia; 7 Department of Gastroenterology and Clinical Nutrition, Royal Children’s Hospital, Melbourne, VIC Australia; 8 Children’s Cancer Centre, Royal Children’s Hospital, Melbourne, VIC Australia; 9 Royal Children’s Hospital, Melbourne, VIC Australia; 10 Department of Biochemistry, Vanderbilt University, Nashville, TN 37232-7917 USA; 11 Department of Medical Genetics, University of Calgary, Calgary, AB Canada; 12 Alberta Children’s Hospital, Section of Pediatric Gastroenterology, Hepatology and Nutrition, University of Calgary, Calgary, AB Canada; 13 Department of Community Pediatrics, University of Calgary, Calgary Alberta, AB Canada; 14 Division of Human Genetics, Cincinnati Children’s Hospital Medical Center, Cincinnati, OH USA; 15 Department of Pediatrics, University of Cincinnati Medical Center, Cincinnati, OH USA; 16 Division of Clinical Genetics, Children’s Mercy Kansas City, Kansas City, MO USA; 17 Department of Pediatrics, University of Missouri—Kansas City School of Medicine, Kansas City, MO USA; 18 Department of Clinical Genetics, Aarhus University Hospital, Aarhus, Denmark; 19 Department of Clinical Medicine, Health, Aarhus University, Aarhus, Denmark; 20 Department of Pediatrics and Adolescent Medicine, Aarhus University Hospital, Aarhus, Denmark; 21 Division of Genetics, Department of Pediatrics, Atrium Health Levine Children’s Hospital, Charlotte, NC USA; 22 Division of Pediatric Gastroenterology, Hepatology and Nutrition at Cincinnati Children’s Hospital Medical Center, Cincinnati, OH USA; 23 Centre for Rare Diseases, Department of Pediatrics and Adolescent Medicine, Aarhus University Hospital, Aarhus, Denmark

**Keywords:** Disease genetics, Molecular biology

## Abstract

DNA repair disorders are a group of conditions characterized by progressive, multisystem phenotypes. Defining new clinical presentations of these disorders is essential for optimizing patient care. ERCC1-XPF is a multifunctional endonuclease involved in nucleotide excision repair (NER) and interstrand crosslink (ICL) repair. We sought to define a novel multisystem phenotype associated with biallelic *ERCC1* variants and impaired DNA repair. Through international collaboration, we identified seven individuals from five families carrying biallelic *ERCC1* variants, including p.Arg156Trp and p.Ala266Pro, who exhibited a distinct clinical phenotype. All individuals presented with growth restriction, photosensitivity, and kidney and liver dysfunction. Notably, three children required liver transplants. Hepatocellular carcinoma developed in four children, resulting in two deaths, including one following treatment with doxorubicin and cisplatin. Older individuals exhibited additional features, including ataxia, basal cell carcinomas, pancreatic insufficiency, ovarian failure, hypothyroidism, and restrictive lung disease. Functional assays using patient-derived fibroblasts demonstrated significant destabilization of the ERCC1-XPF complex and defects in NER and ICL repair. However, residual NER and ICL repair activity was observed, suggesting a hypomorphic effect of the missense variants, which were present either in the homozygous state or in trans with a predicted loss-of-function allele. We define *ERCC1*-hepatorenal syndrome as a severe, multisystem DNA repair disorder associated with high morbidity and mortality, including a significant risk of pediatric hepatocellular carcinoma. We propose management guidelines emphasizing cancer surveillance and caution with chemotherapy to minimize treatment-related toxicity.

## Introduction

DNA repair disorders comprise Fanconi anemia (FA), Xeroderma Pigmentosum (XP) and Cockayne syndrome (CS) [1], and are characterized by pleiotropic phenotypes, including growth restriction, photosensitivity, neurodegeneration (in CS) [2] and susceptibility to malignancies (in XP and FA) [3, 4]. Currently, biallelic variants in 23 genes are involved in FA, all encoding components of the FA repair pathway. Similarly, biallelic variants in 10 genes are involved in XP and CS, all encoding components of the nucleotide excision repair (NER) pathway. Shared between these pathways is ERCC1-XPF, a multifunctional endonuclease involved in NER, interstrand crosslink (ICL) repair, and DNA double-strand break repair [5–11].

Two individuals with CS phenotypes and bi-allelic *ERCC1* (MIM 126380) variants had been previously reported [12, 13]. Both died in early childhood. Cells from both individuals showed sensitivity to UV-induced DNA damage, and individual 165TOR in particular was also mildly sensitive to ICL-inducing agents. The most affected individual, 165TOR [12], carried a premature stop codon (p.Gln158Ter) and a p.Phe231Leu missense variant in *ERCC1*, while the other individual, CS20LO [13], was homozygous for the p.Phe231Leu missense variant. A third individual (XP202DC) with bi-allelic *ERCC1* variants who died at 37 years of age is cited in a meeting abstract [14]. This individual was compound heterozygous for a nonsense variant (K226X) and a splice variant in *ERCC1*. A detailed phenotypic description is not available, and the impact of these *ERCC1* variants is unknown.

In 2021 [15], we reported a family with two affected sisters (PV50LD and PV46LD) who presented with a phenotype overlapping but distinct from CS, FA, and XP. Both carried compound heterozygous *ERCC1* variants: p.Arg156Trp and an exon 4 deletion encompassing the Arg156 residue. Patient-derived fibroblasts showed markedly reduced ERCC1 and XPF protein levels, severely impaired NER activity, and increased chromosome breakage following exposure to DNA cross-linking agents. Both siblings developed liver failure requiring transplantation. These findings suggested that ERCC1 deficiency causes a novel DNA repair disorder.

We now confirm this gene-disease association in a cohort of seven individuals with *ERCC1*-hepatorenal syndrome and expand the phenotype to include multi-organ failure and malignancy risk, particularly childhood-onset hepatocellular carcinoma (HCC). The death of one individual from multisystem organ failure following chemotherapy underscores the heightened sensitivity of individuals with *ERCC1* variants to DNA-damaging agents. The associated cancer risk further highlights the importance of timely molecular diagnosis to guide clinical management and improve outcomes.

## Materials, subjects, and methods

Following the 2021 publication of the original family [15], five additional affected individuals were identified through their clinicians. Each family underwent exome or genome sequencing to determine the molecular cause. This study was part of the Rare Diseases Now project, approved by the Royal Children’s Hospital Ethics Committee (HREC 67401), with protocols also approved by local and regional ethics boards in accordance with national guidelines and the Declaration of Helsinki. Informed consent was obtained from all participants or their guardians, with written consent archived for all patient photographs. Exome sequencing was conducted in four genomic labs using DNA extracted from the whole blood of affected individuals and their parents. Sequencing was performed using standard capture kits per manufacturer's protocols. Parentage was confirmed via inherited variant analysis. All individuals had biallelic *ERCC1* variants. Sequencing methods are detailed in the supplemental material.

### Cell lines

The cell lines used in this study were cultured at 37 °C and 5% CO_2_. Human primary fibroblasts were cultured in Dulbecco’s modified Eagle’s medium (DMEM) supplemented with GlutaMAX™ (Gibco; Thermo Fisher Scientific, Bleiswijk, Netherlands), 20% fetal calf serum (FCS; Thermo Fisher Scientific), and 1% Penicillin/Streptomycin (Pen/Strep). Retinal Pigment Epithelial (RPE1) cell lines were cultured in DMEM supplemented with GlutaMAX™, 10% FCS, and 1% Pen/Strep. Patient fibroblasts were obtained from skin biopsies. All cell lines used are listed in Supplementary Table 1.

### gDNA purification, PCR, and Sanger sequencing

Genomic DNA (gDNA) was extracted from cells and purified as previously described [15, 16]. A 1-kb initial product was amplified from gDNA using primers spanning the missense variant. This product was then PCR amplified again with nested primers (see Supplementary Table 2 for sequencing primers). The product from the second PCR with nested primers was used for Sanger sequencing with the forward primer.

### Western blot

Whole cell lysates were obtained as previously described [15, 16]. Lysates were separated on a 4–12% Criterion XT Bis-Tris gel (Bio-Rad, Veenendaal, Netherlands) and transferred to a PVDF Immobilon membrane (Merck, Amsterdam, Netherlands). To visualize proteins, the membrane was incubated overnight with a primary antibody in blocking buffer, then washed four times with PBS-Tween, and incubated for 1 h in the dark with a secondary antibody (see Supplementary Table 3 for primary and secondary antibodies). Finally, the membrane was washed four times with PBS-Tween, and the Odyssey CLx system (LI-COR, Bad Homburg, Germany) was used to detect proteins. Blot scans were analyzed with Image Studio Lite.

### Immunofluorescence

180,000 cells were seeded on 18 mm coverslips in DMEM (20% FCS and 1% Pen/Strep). Immunofluorescence staining was performed as previously described [15, 16]. Primary antibodies were incubated for 2 h at room temperature in the dark, followed by incubation with secondary antibodies for 1 h and DAPI (Thermo Fisher) for 5 min (see Supplementary Table 3 for primary and secondary antibodies).

### Unscheduled DNA synthesis (UDS)

To measure DNA repair-related DNA synthesis, unscheduled DNA synthesis (UDS) was performed as previously described [16]. See Supplementary Table 3 for primary and secondary antibodies.

### Recovery of RNA synthesis (RRS)

To measure transcription-coupled repair (TCR) activity, Recovery of RNA Synthesis (RRS) was performed as previously described [15, 16]

### MTT assay for cell viability

RPE1 cells (500 cells/well) or primary fibroblasts (1000 cells/well) were seeded in 96-well plates in 100 µL of complete growth medium. After cell attachment, 100 µL of a 2× concentrated compound-containing medium (either Illudin S or MMC) was added to each well, bringing the final volume to 200 µL. Cells were incubated for 7 days at 37 °C in a humidified incubator with 5% CO₂. Following incubation, the medium was removed and wells were washed once with 100 µL PBS. On the final day, 80 µL of DMEM supplemented with 10% FCS was added to each well, followed by 20 µL of MTT solution (Thermo Fisher Scientific, Cat# M6494; 5 mg/mL in PBS). Plates were protected from light and incubated for 1.5 h at 37 °C with gentle shaking. After incubation, the medium was carefully removed and 200 µL DMSO was added to each well to solubilize the purple formazan crystals. Plates were gently mixed at room temperature for 5 min. Absorbance was measured at 570 nm using a plate reader.

### Structural modeling of ERCC1

A structural model of ERCC1 was prepared by combining the PDB coordinates of the ERCC1 central domain bound to XPA (PDB:2JNW), cryo-EM structures of XPF-ERCC1 in apo (PDB: 6SXA) and DNA-bound form (PDB: 6SXB). Variants p.Arg156Trp, and p.Ala266Pro were generated using the mutagenesis wizard in PyMOL Molecular Graphics System, version 2.5.2, Schrödinger. Polar, hydrophobic interactions and clashes were visualized using PyMOL. The PDBePISA server was used to analyze the residues involved at the interaction interfaces between XPF, XPA, and DNA and the energetics of the interactions with and without the variants.

## Results

### Phenotype analysis of clinical cohort with *ERCC1*-hepatorenal syndrome

Following the publication of the original family in 2021 [15], we identified five additional affected individuals through their treating clinicians, all with biallelic *ERCC1* variants confirmed by genomic sequencing. We also provide a clinical update on the two originally reported sisters. The age range of the cohort is 3–18 years. As detailed in Table 1, individuals in this cohort exhibit a multisystem phenotype including significant growth restriction, variable developmental issues, significant skin and ocular photosensitivity, and liver impairment with an onset in infancy, leading to cirrhosis and liver failure. In addition, four individuals developed HCC in childhood. Kidney problems are present in some individuals, often exacerbated by treatment-related factors. These manifestations reveal a new *ERCC1*-hepatorenal syndrome.

**Table 1:** Phenotype information for individuals with *ERCC1* variants.

Individual identifier	PV50LD	PV46LD	CA16LD	XE28CH	XE23CI	XE24CI	XE1AH
Family	1	1	2	3	4	4	5
*ERCC1* variant 1, NM_001983.4 NP_001974.1	c.466 C > T p.Arg156Trp	c.466 C > T; p.Arg156Trp	c.466 C > T; p.Arg156Trp	c.466 C > T; p.Arg156Trp	c.466 C > T; p.Arg156Trp	c.466 C > T; p.Arg156Trp	c.790 G > C p.Ala266Pro
*ERCC1* variant 2, NM_001983.4 NP_001974.1	c.321+61_525+132del p?	c.321+61_525+132del p?	c.466 C > T; p.Arg156Trp	c.703-2 A > G p?	c.525+2 T > C p?	c.525+2 T > C p?	c.790 G > C p.Ala266Pro
Sex	Female	Female	Male	Female	Male	Male	Female
Deceased?	no	no	no	yes	yes	No	no
Age at last assessment	18 y	15 y 10 m	3 y 11 m	11 y 9 m	8 y 4 m	11 y 7 m	16 y 10 m
Weight, centile Height, centile OFC, centile	28 kg ( < 1st, Z-8.8) 139 cm (Z-3.72) NR	24 kg ( < 1s, Z-10) 135 cm ( < 1st, Z-4.2) NR	8.745 kg ( < 1st) 77.5 cm ( < 1st) 46.2 (7^th^)	20.8 kg ( < 1st) 129 cm ( < 1st) 49.5 cm	20.5 kg (2.7^th^) 130.1 cm (53^rd^) 45.7 cm at 35 months -2.3 sd)	27.1 cm (2^nd^) 142 cm (29^th^) 48.5 cm (-3.5 sd)	42.5 kg (-2.5 SD) 158 cm (-1.5 SD) 53 cm (- 2 SD)
Dysmorphic features	Deep-set eyes Micrognathia	Deep-set eyes Retrognathia	Short palpebral fissures	Deep-set eyes	None reported	Triangular-shaped face, deep-set eyes	Petite facial features (small face and small, pointed nose)
Liver	Cholestatic liver dysfunction at 18 months. Progressed to liver failure and transplant aged 9 y	Liver impairment from 2 years, liver biopsy showed moderate number of double-nucleated hepatocytes, some with large nuclei and nucleoli Progressed to liver failure and transplant aged 8 y	Neonatal cholestasis which resolved, then developed mild cholestatic hepatitis with normal synthetic function. Acute rise in AFP at 3 y 4 m with suspicious lesions identified on CEUS. Biopsies were consistent with HCC. The patient is undergoing transplant evaluation at time of manuscript preparation	Progressive cholestatic liver dysfunction at age 9 y Metastatic multifocal HCC diagnosed leading to death aged 11 y 9 m	Cirrhotic liver with multiple nodules on presentation, biopsy showed HCC, macro-trabecular subtype. Acute on chronic liver failure with increasing liver transaminases/coagulopathy/encephalopathy after 1st cycle of chemotherapy. Died from multi-system organ failure aged 8 y 4 m	Surveillance due to brother’s HCC showed rising AFP and liver MRI showed suspicious lesions. Biopsies confirmed moderately differentiated multifocal HCC. Received liver transplant at 10 y 11 m	Cryptogenic cirrhosis with bile duct proliferation, mild inflammation and degenerative changes, suggestive of chronic cholestatic disease. MRCP with multiple hypointense focal liver lesions, progressive in size and number (possibly dysplastic noduli) and stenosis in distal part of common hepatic duct
Kidney	Proximal tubular dysfunction with albuminuria and hypercalciuria. Kidney function fluctuated, with intermittent episodes of acute kidney injury, progressive kidney impairment, with increasing creatinine levels and minimal response to acetyl cholinesterase inhibition. Kidney ultrasound showed small echogenic kidneys with reduced corticomedullary differentiation. Progressed to stage 5 chronic kidney disease and is under consideration for a kidney transplant	Renal tubulopathy with mild kidney impairment, with progressively increasing creatinine levels. Ultrasound showed small kidneys with nephrocalcinosis. Progressed to stage 5 chronic kidney disease and is under consideration for a kidney transplant.	Neonatal nephrocalcinosis, which resolved	Recurrent UTIs; Kidney dysfunction (Glucosuria, proteinuria, hyperphosphaturia, metabolic acidosis, and hypophosphatemia - consistent with Fanconi renotubular phenotype)	Stage 3 AKI secondary most likely to cisplatin. Consistent with nephrotoxicity but greater than expected. Kidney biopsy: Severe tubular injury with reactive focal nuclear enlargement and mild glomerular injury with no definitive glomerulonephritis.	Mild impairment Developed hypertension post-liver transplant	Normal kidney ultrasound, BP and creatinine, but low free carnitine and glucosuria, suggestive of proximal tubular kidney dysfunction; further evaluation pending
Skin	Freckling on sun-exposed areas. Six BCCs removed in teenage years	Freckling on sun-exposed areas. Scalp BCC removed	Numerous café-au-lait macules. Severe photosensitivity	Numerous freckles Significant. photosensitivity	Malar freckling	Numerous café-au-lait macules Severe photosensitivity	Freckling on sun-exposed areas Photosensitivity Café-au-lait macules
Ocular photosensitivity	yes	yes	yes	yes	Not reported	Not reported	yes
Development	Mild intellectual disability, IQ 68	Mild intellectual disability, IQ63	Mild developmental delay	Normal	Learning difficulties ADHD	Intellectual disability	Normal
Brain MRI	Mild cerebral atrophy with moderate cerebellar atrophy and mild brainstem atrophy (age 12 y)	Normal MRI at 5 y. MRI age 10 y showed moderate cerebellar atrophy and mild cortical atrophy	Normal MRI at 15 m	NR	NR	NR	NR
Other	Pancreatic insufficiency Type 1 Diabetes mellitus Hypothyroidism Ovarian failure Ataxia, tremor Moderate-severe restrictive lung disease Kyphosis	Exocrine pancreatic insufficiency Ovarian failure Hypothyroidism Ataxia, tremor		Myopia Amblyopia	Steroid-induced diabetes	Motor tics, gait ataxia Post-liver transplant developed Type 1 diabetes mellitus and esophageal candidiasis	Primary ovarian insufficiency Missing permanent teeth Thenar hypoplasia Restricted range of movement in thumbs bilaterally Recurrent lower airway infections and decreased FEV1 and FVC with negative reversibility test; further evaluation pending Motor tics

All individuals exhibit significant growth restriction. Where available, birth growth parameters for weight, head circumference and length were typically under the first centile or at the low end of the normal range. Postnatally, individuals demonstrated faltering growth, although this varied in severity and across growth domains. In the originally reported siblings, the older sister had an adult height of 139 cm (*Z* = −4), while the younger sister had a current height of *Z* = −4 at age 15. Both showed extremely low weight, with Z-scores of −8 and −10, respectively. Developmental milestones were normal in 5/7 individuals with two exhibiting delay. The older individuals in the cohort had learning difficulties or mild intellectual disability. Skin and ocular photosensitivity is a notable feature, with 6/7 individuals experiencing skin and/or ocular reactions to sun exposure, including sunburn requiring hospitalization and severe corneal burns. These symptoms are consistent with the known link between *ERCC1* defects and impaired DNA repair [11]. Although no skin cancers were reported initially [15], since publication, individual PV50LD has had six basal cell carcinomas removed from sun-exposed areas during adolescence, and PV46LD had one removed from the scalp at age 11. Two other individuals in the cohort also presented with multiple café-au-lait macules from infancy.

**Fig. 1:**
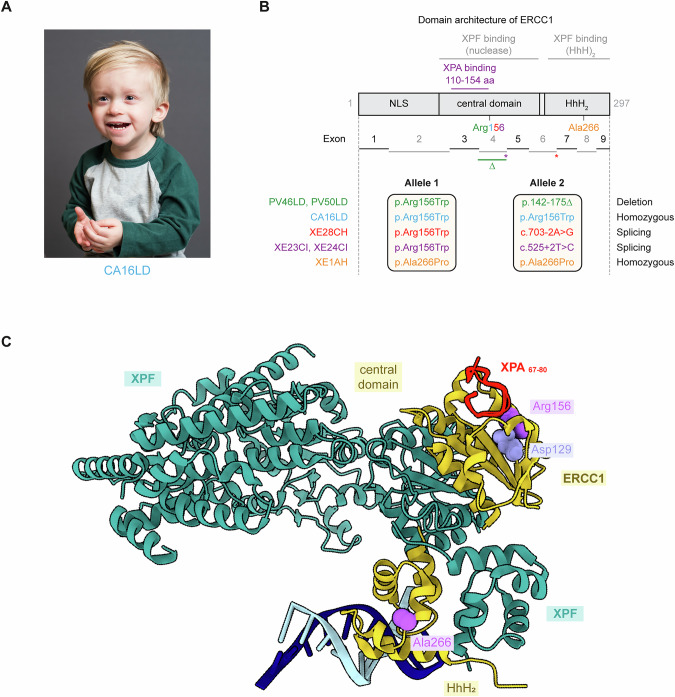
A small cohort of patients with *ERCC1*-hepatorenal syndrome. **A** Facial photographs of individual CA16LD demonstrating short palpebral fissures and petite facial features. **B** Schematic representation of the ERCC1 protein. The interaction sites of ERCC1 with other proteins are depicted above. Known variants in the protein, including the variant in individual CA16LD (highlighted in blue), are depicted below. The variants in the other individual in our cohort are also indicted. **C** Cryo-EM structure of the ERCC1-XPF heterodimer bound to DNA, based on 6SXB. The XPA peptide (in red, from 2JNW) is superimposed on the cryo-EM structure. Both Arg156 and Ala266 are indicated.

Liver involvement was observed in all individuals, with age-dependent onset and variable severity. PV50LD and PV46LD developed progressive cholestatic liver disease, requiring liver transplants at ages 9 and 8, respectively [15]. Prior to transplant, a biopsy of PV46LD showed intrahepatic bile duct damage, periductal fibrosis, and numerous double-nucleated hepatocytes. XE28CH developed abnormal liver function at age 9, progressing to cirrhosis and metastatic HCC; she died at age 11. XE23CI also developed cirrhosis, and following a raised alpha-fetoprotein (AFP), was diagnosed with HCC. Shortly after starting chemotherapy, he developed coagulopathy, encephalopathy, cholestatic liver failure, and profound bilateral hearing loss, and died from multisystem organ failure. Due to his brother’s diagnosis, XE24CI underwent regular MRI surveillance, which revealed fibrotic liver changes and a suspicious lesion with elevated AFP. Biopsy confirmed HCC, and he received a liver transplant. CA16LD (Fig. 1A) had mildly elevated liver enzymes from 6 months of age. At age 2, ultrasound revealed a stable echogenic lesion, likely a hemangioma. AFP levels were mildly elevated from 2 years and 8 months, with a sharp rise at 3 years and 4 months. Contrast-enhanced ultrasound (CEUS) identified two additional lesions, one suspicious for HCC. Although MRI was inconclusive, biopsy confirmed HCC. He is currently being evaluated for liver transplantation. At age 14, XE1AH had mildly elevated ALT and a normal liver ultrasound. By age 17, she had developed cirrhosis and early esophageal varices. Across the cohort, liver disease ranged from mild enzyme elevation to cirrhosis, acute-on-chronic liver failure, and HCC.

Kidney involvement was variable and generally milder. CA16LD had nephrocalcinosis with preserved kidney function. XE23CI developed acute kidney injury due to cisplatin toxicity, requiring hemodialysis. XE24CI had mild impairment before liver transplantation and was managed with kidney-sparing immunosuppression. PV50LD and PV46LD developed tubular dysfunction by age six, which fluctuated but led to progressive renal impairment and reduced creatinine clearance. Ultrasound showed small kidneys with poor corticomedullary differentiation in both, and nephrocalcinosis in PV46LD. Both individuals progressed to stage 5 chronic kidney disease and are currently being evaluated for transplantation.

Two individuals died in the first decade, one from metastatic HCC, the other from multisystem failure following chemotherapy. Among those over age ten, additional complications emerged, reflecting multisystem involvement: type 1 diabetes, exocrine pancreatic insufficiency, hypothyroidism, ovarian failure, restrictive lung disease, and ataxia. Four individuals showed abnormal chromosome breakage in response to Mitomycin C or diepoxybutane, consistent with the involvement of ERCC1 in ICL repair.

### Variant analysis

The five *ERCC1* variants identified in this cohort are summarized in Supplementary Table 4. Six of the seven individuals carried the p.Arg156Trp substitution on one allele, paired with either a splice variant (XE28CH, XE23CI, XE24CI), a deletion (PV46LD, PV50LD), or a second p.Arg156Trp variant (CA16LD) (Figs. 1B, S1A). One individual (XE1AH) was homozygous for the p.Ala266Pro variant.

Both missense variants, p.Arg156Trp and p.Ala266Pro, affect highly conserved residues and have AlphaMissense scores in the likely pathogenic range (0.96 and 0.98, respectively). Arginine 156 lies in the middle domain of ERCC1 near the XPA-binding pocket and is thought to stabilize domain structure (Figs. 1C, S1B) [17, 18]. Substitution with tryptophan is predicted to disrupt interactions with Tyr152 and Asp129 (Fig. S2), destabilizing the domain and reducing ERCC1 protein stability [15, 19]. Alanine 266 is located at a structural kink in the (HhH)₂ domain between the XPF-interacting region and the C-terminal helix that binds dsDNA (Figs. 1C, S3). Substitution to proline likely alters domain conformation and dynamics, impairing DNA binding and interaction with the XPF (HhH)₂ domain.

The p.Arg156Trp variant appears in heterozygous form in 204 individuals in GnomAD v4 [20], with no homozygotes. A significant difference in allele frequency between exome and genome data suggests possible technical artifacts in exome calling. One individual in GnomAD v4 (non-UK Biobank) is homozygous for p.Ala266Pro and is under 30 years of age, although no further data are available. The two canonical splice variants, c.703-2 A > G and c.525+2 T > C, are predicted to disrupt splicing, with SpliceAI scores of 0.98 and 0.99, respectively, and likely result in nonsense-mediated decay. The exon 4–5 deletion removes most of the central domain and is predicted to produce a null allele.

### The *ERCC1* p.Arg156Trp and p.Ala266Pro substitutions destabilizes ERCC1-XPF

**Fig. 2:**
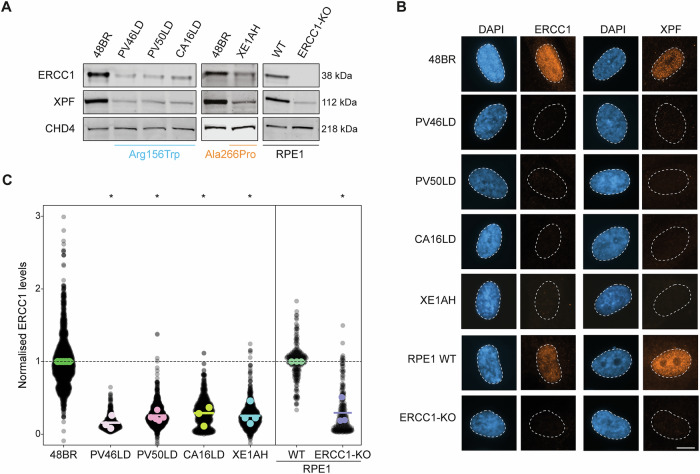
CA16LD and XE1AH fibroblasts have low steady-state levels of ERCC1-XPF. **A** Western blot showing protein expression of ERCC1 and XPF in 48BR (WT), patient fibroblasts PV46LD, PV50LD, and CA16LD carrying the p.Arg156Trp variant, XE1AH carrying the p.Ala266Pro variant, or RPE1-hTERT cells that are either WT or KO for *ERCC1*. Levels of CHD4 are shown as a loading control. **B** Representative microscopic images and **C** quantification of the intensity of immunofluorescently labeled ERCC1 in the nuclei (stained with DAPI) of PV46LD, PV50LD, and CA16LD cells, as well as in wildtype (48BR and RPE1) and *ERCC1*-KO. Protein levels in patient fibroblasts were normalized to control fibroblasts 48BR, and levels in RPE1 *ERCC1*-KO were normalized to those in RPE1 WT. Each datapoint represents one cell, with the colored bar depicting the mean of all datapoints, and the colored points representing the mean of the individual replicates. The scale bar is 10 µm. Statistical significance was determined by a paired 2-tailed t-test (* = *p* < 0.05).

For functional testing, primary fibroblasts were obtained from skin biopsies of individuals CA16LD (Fig. 1A) and XE1AH. Sanger sequencing confirmed the homozygous c.466 C > T (p.Arg156Trp) variant in CA16LD and the homozygous c.796 G > C (p.Ala266Pro) variant in XE1AH (Fig. S1A). The p.Arg156Trp variant was also present on one allele in fibroblasts from PV46LD and PV50LD [15], which were analyzed in parallel. To assess the impact on ERCC1 protein levels, western blotting was performed. Given XPF stability depends on ERCC1, XPF levels were also measured. Fibroblasts from CA16LD, XE1AH, PV46LD, and PV50LD showed reduced ERCC1 levels compared to control (48BR), although ERCC1 remained detectable, unlike in the ERCC1 knockout (KO) RPE1 cell line [15]. XPF levels were similarly reduced across all patient fibroblasts, comparable to the ERCC1-KO line (Fig. 2A). XE1AH cells also showed marked reduction in both proteins, suggesting the p.Ala266Pro variant destabilizes the ERCC1-XPF heterodimer.

To confirm and quantify these findings, ERCC1 and XPF were visualized by immunofluorescence in fixed cells. Nuclear levels of both proteins were markedly reduced in CA16LD and XE1AH fibroblasts compared to wild-type (48BR), with PV46LD and PV50LD showing similar staining (Fig. 2B). Quantification revealed ERCC1 and XPF levels in CA16LD and XE1AH were reduced to ~30%, similar to PV50LD (Figs. 2C, S4). Detected protein levels in *ERCC1*-KO RPE1 cells were within the same range. Both western blot and immunofluorescence confirmed reduced ERCC1 and XPF levels in cells with either the p.Arg156Trp or p.Ala266Pro variant, consistent with predicted destabilization. Protein levels were consistent across all patient fibroblasts, indicating that CA16LD and XE1AH cells behave similarly to previously reported cases (Fig. 2B, C).

### CA16LD and XE1AH fibroblasts exhibit a strong NER defect

**Fig. 3:**
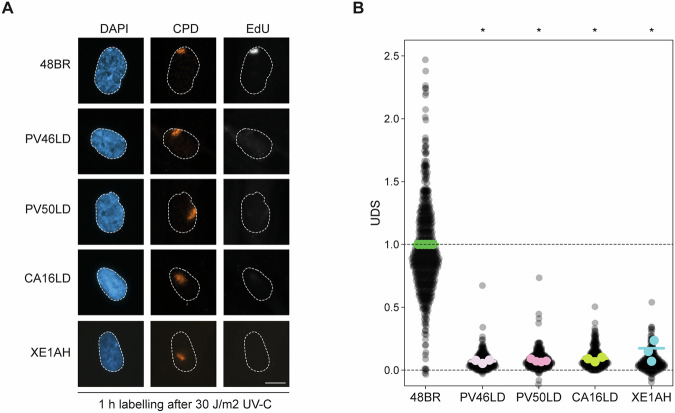
CA16LD and XE1AH fibroblasts show a defect in GGR function. **A** Representative microscopic images of nuclei (DAPI), local UV damage (CPD), and unscheduled DNA synthesis (EdU), and **B** quantification of the EdU intensity at the site of local damage in ERCC1 patient fibroblasts after local UV-C irradiation. EdU is incorporated for 1 h after 30 J/m² UV irradiation. The intensity of EdU is normalized to wildtype 48BR. Each datapoint represents one local damage site, with the colored bar depicting the mean of all datapoints, and the colored points representing the mean of the individual replicates. The scale bar is 10 µm. Statistical significance was determined by a paired 2-tailed t-test (* = *p* < 0.05).

To assess the impact of the p.Arg156Trp and p.Ala266Pro variants and the resulting reduction in ERCC1-XPF levels on NER, we evaluated both global genome repair (GGR) and TCR activity. GGR, which accounts for up to 95 percent of NER activity, was measured by UDS following local UV irradiation [21]. Cells were incubated with the thymidine analog EdU for one hour to label repair-associated DNA synthesis. EdU incorporation at UV-damaged sites, identified by CPD staining, was significantly reduced in *ERCC1* patient fibroblasts compared to wild-type 48BR cells. Quantification confirmed impaired GGR in CA16LD and XE1AH, with levels similar to PV46LD and PV50LD [15] (Figs. 3A, B; S5).

**Fig. 4:**
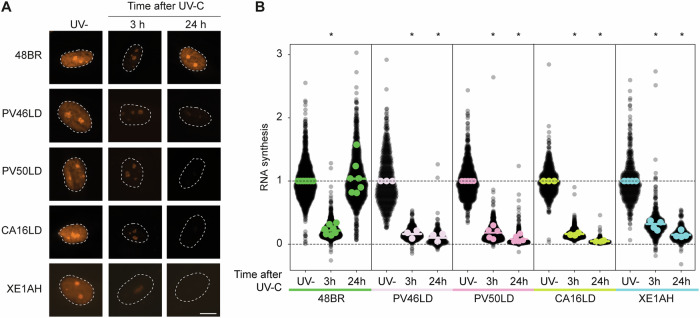
CA16LD and XE1AH fibroblasts are deficient in TCR. **A** Representative microscopic images of the incorporation of EU in nuclei (identified with DAPI), and **B** quantification of the EU incorporation for 48BR (WT) and ERCC1 patient fibroblasts. EU was incorporated for 1 h at different time points after 16 J/m² UV-C irradiation (mock, 3  and 24 h). For each cell line, data were normalized to the mock-treated condition. Each datapoint represents one cell, with the colored bar depicting the mean of all datapoints, and the colored points representing the mean of the individual replicates. The scale bar is 10 µm. Statistical significance was determined by a paired 2-tailed t-test (* = *p* < 0.05).

To further assess NER, we examined TCR, which repairs transcription-blocking lesions [22]. RRS was measured using the uridine analog EU at 0, 3, and 24 h after UV irradiation. At 3 h, EU incorporation was markedly reduced in all lines. By 24 h, 48BR cells showed recovery to near-baseline levels, while CA16LD, XE1AH, PV46LD, and PV50LD exhibited persistently low EU labeling. Quantification confirmed reduced RNA synthesis at 3 h in all lines, with only 48BR showing recovery at 24 h. These results indicate defective TCR in all *ERCC1* patient fibroblasts (Fig. 4A, B).

### CA16LD and XE1AH fibroblasts are sensitive to DNA-damaging agents

To further investigate the impact of defective DNA repair in *ERCC1*-deficient fibroblasts, we assessed cell viability and metabolic activity using the MTT assay after seven days of exposure to DNA-damaging agents Illudin S or Mitomycin C (MMC). Illudin S induces transcription-blocking lesions repaired by TCR, while MMC causes ICLs repaired through a distinct replication-coupled pathway involving the FA complex [9, 23–25]. Unlike proteins specific to either NER or FA repair, ERCC1-XPF functions in both pathways.

**Fig. 5:**
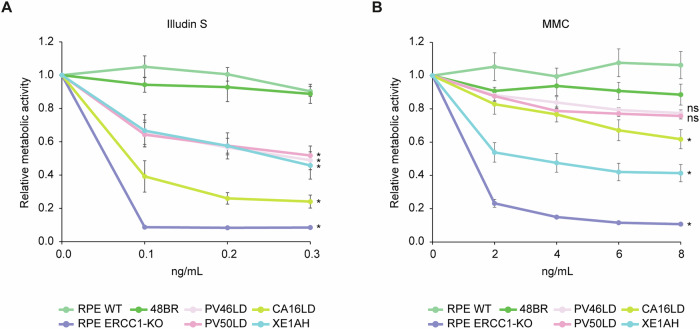
Fibroblasts from individual CA16LD and XE1AH are sensitive to Illudin S and Mitomycin C. MTT assay following exposure to **A** Illudin S, and **B** MMC to measure viability of the indicated cell-lines at 7 days. Statistical significance was determined by a paired 2-tailed t-test (* = *p* < 0.05) between cell-lines and matching control.

Following Illudin S exposure, PV46LD, PV50LD, and XE1AH fibroblasts showed intermediate sensitivity, while CA16LD cells were more sensitive. However, none were as sensitive as *ERCC1*-KO RPE1 cells included as a control (Fig. 5A). This indicates a strong TCR defect in patient cells, although residual repair activity likely reduces sensitivity to transcription-blocking damage, consistent with previous UV response findings [15]. After MMC exposure, CA16LD cells (p.Arg156Trp) showed mild sensitivity, while XE1AH cells (p.Ala266Pro) were more sensitive. Still, none matched the hypersensitivity of *ERCC1*-KO RPE1 cells (Fig. 5B). These results align with earlier findings showing mild ICL repair defects in PV46LD and PV50LD [15], and are supported by chromosome breakage observed in CA16LD cells after MMC treatment.

Together, these findings suggest that residual NER and ICL repair activity may partially mitigate the full clinical manifestations observed in disorders, such as XP or FA, likely due to the hypomorphic effects of the missense variants present in this cohort. We propose that the liver and kidney phenotypes in these patients result from high DNA damage burden in these organs, due to combined deficiencies in transcription- and replication-coupled repair involving ERCC1-XPF, a unique feature of this multifunctional nuclease.

## Discussion

We report a cohort with a multisystem phenotype characteristic of a DNA repair disorder, including growth restriction, café-au-lait macules, and photosensitivity, along with notable findings of kidney impairment, progressive cholestatic liver disease, and HCC. Older individuals also showed multi-organ involvement. Pediatric-onset progressive liver disease is a distinctive feature of this cohort and has not been previously reported in individuals with XP-F or Fanconi syndrome [26, 27]. Six of the seven individuals carry at least one copy of the c.466 G > A (p.Arg156Trp) *ERCC1* variant. CA16LD is homozygous for p.Arg156Trp, while XE28CH, XE23CI, and XE24CI are compound heterozygotes with a splice variant on the second allele, predicted to undergo nonsense-mediated decay. XE1AH is homozygous for the c.796 G > C (p.Ala266Pro) variant. Although this variant appears in homozygous form in one individual in GnomAD, other variant metrics support its pathogenicity. Given the age-dependent nature of the phenotype, it is plausible that this individual could be affected by *ERCC1*-hepatorenal syndrome but included in the database due to young age at phenotyping. Alternatively, the variant may be associated with a milder phenotype that escapes clinical detection. Notably, XE1AH developed liver disease later than others in the cohort.

Detailed structural modeling showed that the Arg156 side chain forms a hydrogen bond with the hydroxyl group of Tyr152, a stabilizing element that also mediates contacts with XPA (Fig. S2) [17, 18]. Additionally, Arg156 forms a salt bridge with Asp129 (Figs. 1C, S1B). Substitution of Arg156 with Trp is predicted to disrupt both the hydrogen bond with Tyr152 and the salt bridge with Asp129, destabilizing the domain. This provides a structural explanation for the reduced ERCC1 stability observed in patient cells [15, 17, 19]. The c.466 G > A variant lies in exon 4, within the central domain of ERCC1 and near the XPA interaction site. Structural modeling also provided insight into the p.Ala266Pro variant. Cryo-EM structures of the ERCC1-XPF heterodimer suggest that the (HhH)₂ domains of ERCC1 and XPF must undergo significant rearrangement to transition from an autoinhibited state to a DNA-bound configuration [28]. Substitution of Ala266 with Pro introduces conformational constraints that likely impair these dynamic changes, affecting DNA binding and destabilizing interactions with the XPF (HhH)₂ domain. Consistent with these predictions, western blotting showed significantly reduced ERCC1 and XPF protein levels in fibroblasts from CA16LD (p.Arg156Trp) and XE1AH (p.Ala266Pro). Immunofluorescence revealed approximately 30% of normal protein levels in CA16LD, similar to PV46LD and PV50LD.

Fibroblasts from CA16LD (p.Arg156Trp) and XE1AH (p.Ala266Pro) showed impaired GGR and TCR, consistent with findings in PV46LD and PV50LD, who also carry the p.Arg156Trp variant [15]. The residual GGR activity in CA16LD may explain the milder phenotype compared to more severe *ERCC1*-related cases, such as 165TOR [12] and CS20LO [13], both of whom presented with features of CS. Individual 165TOR, compound heterozygous for a nonsense variant (Q158X) and a missense variant (F231L) in the (HhH)₂ motif, had growth retardation, severe developmental delay, contractures, and died in infancy from pneumonia. CS20LO, homozygous for F231L, had contractures, microcephaly, and hypertonia, and died in the second year of life. However, residual repair activity alone may not fully explain the phenotypic differences, suggesting that additional factors or functions of ERCC1-XPF contribute to disease severity.

Unlike NER-deficient mice [29], mice with mutations in *Ercc1* or *Xpf* develop severe liver dysfunction and die from liver failure [11, 30–32]. The liver phenotype in individuals with *ERCC1*-hepatorenal syndrome closely mirrors these models, including accelerated liver aging, hepatocellular senescence, fibrosis, increased polyploidy, apoptosis, and reduced regenerative capacity [33, 34]. However, early-onset HCC, as seen in our cohort, has not been reported in mice. Notably, liver-specific restoration of *Ercc1* in deficient mice improved liver function and extended lifespan, but these mice later died of renal failure, with polyploidy observed in kidney tubule cells [35]. This suggests that kidney dysfunction is not secondary to liver disease but reflects a direct, cell-intrinsic role for ERCC1 in renal protection.

Renal involvement in our patient cohort is variable, including tubular dysfunction and nephrocalcinosis. The two oldest individuals progressed to end-stage renal disease in adolescence. This renal phenotype resembles that seen in CS [36], but contrasts with XP, where kidney involvement is not typical. Similarly, *Ercc1*^*-/Δ7*^ mice show glomerulosclerosis and tubular atrophy, consistent with accelerated renal aging [34, 37]. Mice with podocyte-specific *Ercc1* deletion also develop glomerular damage, podocyte loss, and proteinuria [38, 39]. These findings support monitoring for intrinsic kidney dysfunction in *ERCC1*-hepatorenal syndrome, especially post-liver transplant. Future studies could explore the tissue-specific effects of the p.Arg156Trp variant in mouse liver.

The origin of the DNA damage driving liver and kidney pathology remains unclear. Rather than a single lesion type, we propose that a high burden of diverse DNA lesions accumulates in these organs due to combined defects in transcription- and replication-coupled repair involving ERCC1-XPF. For example, *Xpa*/*Fanca* double-KO mice, deficient in both NER and ICL repair, do not develop liver disease [29], suggesting other ERCC1-dependent pathways may offer protection. However, *Adh5*^-/-^ *Fancd2*^-/-^ mice, which accumulate high levels of endogenous ICLs, do develop liver dysfunction and cancer [40]. Conversely, *Adh5*^-/-^ *Csb*^-/-^ mice, deficient in TCR, develop kidney but not liver failure [41], suggesting the liver may rely more on replication-coupled repair, while the kidney depends on TCR. Mouse models thus recapitulate key features of *ERCC1*-hepatorenal syndrome, although early-onset HCC and end-stage renal disease remain unique to humans, possibly due to species-specific or environmental factors.

While there is some overlap between the phenotype in this cohort and other DNA repair disorders, such as CS and XP, key features, including early-onset liver disease, a high incidence of pediatric HCC, and renal dysfunction, distinguish this syndrome and support the need for specific clinical management guidelines. The *ERCC1*-hepatorenal phenotype overlaps with the single reported case of a 15-year-old boy with biallelic *XPF* mutations (XP51RO; p.R153P; c.458 G > C) and a progeroid presentation [42]. Shared features include photosensitivity without skin cancer, short stature, loss of subcutaneous fat, developmental delay, and renal insufficiency. However, there are important differences. XP51RO had normal birth growth parameters and presented with growth concerns and a progeroid appearance during adolescence, whereas our cohort typically presented at birth or shortly thereafter with growth restriction. Additionally, facial features of premature aging were generally less prominent in our cohort than those described in XP51RO. Moreover, XP51RO also exhibited only mild liver involvement, without cholestasis or HCC [12, 42]. Identifying additional individuals with *XPF* variants is necessary to determine whether these two phenotypes represent a spectrum or distinct clinical entities. Accurately defining these syndromic boundaries is essential to ensure appropriate clinical care and surveillance.

This study provides a detailed characterization of the *ERCC1*-hepatorenal syndrome phenotype. The progressive, multisystem nature of the condition highlights the importance of early and accurate molecular diagnosis to enable timely monitoring and treatment of organ involvement. UV protection is recommended due to photosensitivity and the underlying DNA repair defect. Regular liver function monitoring and imaging are essential. Only two individuals in our cohort are alive without liver transplantation: CA16LD, aged three, currently being assessed for liver transplant; and XE1AH, aged 17, with progressive cirrhotic liver disease. Based on clinical observations, we propose management guidelines for newly diagnosed individuals (Table 2). Given the rarity of pediatric HCC, surveillance strategies are adapted from adult protocols. We recommend ultrasound and AFP monitoring every three to six months, which are accessible and do not require sedation. Annual MRI is advised from diagnosis, with earlier imaging if ultrasound findings, AFP elevation, or cirrhosis are present. CEUS may also aid lesion characterization without radiation exposure. Further data are needed to refine surveillance intervals and assess HCC risk. If HCC is diagnosed, treatment options include surgical resection, liver transplantation, and systemic therapies such as chemotherapy or targeted agents like sorafenib [43–45]. Complete resection or transplantation remains the primary determinant of survival. Systemic therapies may be used pre- or post-operatively, but outcomes for unresectable or metastatic HCC remain poor. The role of systemic therapy in children with resectable disease is still evolving. In *ERCC1*-hepatorenal syndrome, chemotherapy must be used cautiously due to increased toxicity from DNA repair defects. Platinum-based agents like cisplatin pose particular risks. Given the high risk of decompensation and frequent liver involvement, early consideration of liver transplantation is warranted. In our cohort, all transplanted individuals survived up to nine years post-transplant.

**Table 2:** Clinical care guidelines for individuals with *ERCC1*-hepatorenal syndrome (Unless specified, all surveillance recommendations are suggested from the time of diagnosis).

System	Evaluation	Frequency	Comments
Liver	Liver function tests Abdominal ultrasound (US) Alpha feto protein (AFP) MRI and/or CEUS if available	Annual 3 months 3 months Annual from time of diagnosis. Also consider MRI or CEUS if a lesion is seen on US or there is a rise in AFP, or in presence of cirrhosis	Refer to hepatologist if liver function abnormal
Kidney	Kidney ultrasound to assess growth, echogenicity, and the presence of nephrocalcinosis. Comprehensive urinalysis to screen for albuminuria, low molecular weight proteinuria, electrolyte imbalances, and glucosuria. Serum biochemistry, including measurements of urea, creatinine, and electrolytes. Blood pressure monitoring to identify hypertensive complications. Individuals should be considered as having reduced kidney reserve and nephrotoxic agents should be avoided where alternatives exist, e.g., if choosing antibiotics to treat infection.	Annual	Refer to nephrologist if abnormal
Growth	Document height, weight and head circumference Early referral to a dietitian to optimize weight gain	Annual	
Neurological function	Monitoring of development Consider a brain MRI if regression occurs	Annual	
Skin	Rigorous avoidance of sun and other UV exposures, similar to the management of individuals with XP Examination of skin by a dermatologist	Annual	Refer to a dermatologist
Eyes	Rigorous avoidance of ocular exposure to sun – sunglasses Examination of eyes by an ophthalmologist	Annual	Refer to an ophthalmologist
Thyroid	Thyroid function tests	Annual	
Puberty	Clinical monitoring of pubertal development	Annual from age ten years	
Respiratory function	Clinical monitoring of respiratory symptoms and consideration of lung function testing	Annual from ten years	
Pancreatic function	Fasting blood glucose Fecal elastase	Annual Annual	

In conclusion, we describe a cohort with *ERCC1*-hepatorenal syndrome, characterized by progressive cholestatic liver disease, early-onset HCC, renal impairment, photosensitivity, and growth restriction. We provide clinical management recommendations and emphasize the need for caution with chemotherapy. Ongoing follow-up is essential to better understand the long-term course and optimize care for affected individuals.

## Supplementary information

supplemental information

## Data Availability

The data generated or analyzed during this study can be found within the published article and its supplementary files. Raw data files are available from the corresponding author on reasonable request. Deidentified patient data can be made available on request to the corresponding author.

## References

[1] Keijzers G, Bakula D, Scheibye-Knudsen M. Monogenic diseases of DNA repair. New Engl J Med. 2017;377:1868–76. 29117491 10.1056/NEJMra1703366

[2] Calmels N, Botta E, Jia N, Fawcett H, Nardo T, Nakazawa Y, et al. Functional and clinical relevance of novel mutations in a large cohort of patients with Cockayne syndrome. J Med Genet. 2018;55:329–43. 29572252 10.1136/jmedgenet-2017-104877

[3] Kraemer KH, Patronas NJ, Schiffmann R, Brooks BP, Tamura D, DiGiovanna JJ. Xeroderma pigmentosum, trichothiodystrophy and Cockayne syndrome: a complex genotype-phenotype relationship. Neuroscience. 2007;145:1388–96. 17276014 10.1016/j.neuroscience.2006.12.020 PMC2288663

[4] Ceccaldi R, Sarangi P, D’Andrea AD. The Fanconi anaemia pathway: new players and new functions. Nat Rev Mol Cell Biol. 2016;17:337–49. 27145721 10.1038/nrm.2016.48

[5] Manandhar M, Boulware KS, Wood RD. The ERCC1 and ERCC4 (XPF) genes and gene products. Gene. 2015;569:153–61. 26074087 10.1016/j.gene.2015.06.026 PMC4536074

[6] Adair GM, Rolig RL, Moore-Faver D, Zabelshansky M, Wilson JH, Nairn RS. Role of ERCC1 in removal of long non-homologous tails during targeted homologous recombination. Embo J. 2000;19:5552–61. 11032822 10.1093/emboj/19.20.5552 PMC313999

[7] Ahmad A, Robinson AR, Duensing A, van Drunen E, Beverloo HB, Weisberg DB, et al. ERCC1-XPF endonuclease facilitates DNA double-strand break repair. Mol Cell Biol. 2008;28:5082–92. 18541667 10.1128/MCB.00293-08 PMC2519706

[8] de Laat WL, Appeldoorn E, Jaspers NG, Hoeijmakers JH. DNA structural elements required for ERCC1-XPF endonuclease activity. J Biol Chem. 1998;273:7835–42. 9525876 10.1074/jbc.273.14.7835

[9] Klein Douwel D, Boonen RA, Long DT, Szypowska AA, Raschle M, Walter JC, et al. XPF-ERCC1 acts in unhooking DNA interstrand crosslinks in cooperation with FANCD2 and FANCP/SLX4. Mol Cell. 2014;54:460–71. 24726325 10.1016/j.molcel.2014.03.015 PMC5067070

[10] de Laat WL, Sijbers AM, Odijk H, Jaspers NG, Hoeijmakers JH. Mapping of interaction domains between human repair proteins ERCC1 and XPF. Nucleic Acids Res. 1998;26:4146–52. 9722633 10.1093/nar/26.18.4146 PMC147836

[11] Sijbers AM, de Laat WL, Ariza RR, Biggerstaff M, Wei YF, Moggs JG, et al. Xeroderma pigmentosum group F caused by a defect in a structure-specific DNA repair endonuclease. Cell. 1996;86:811–22. 8797827 10.1016/s0092-8674(00)80155-5

[12] Jaspers NG, Raams A, Silengo MC, Wijgers N, Niedernhofer LJ, Robinson AR, et al. First reported patient with human ERCC1 deficiency has cerebro-oculo-facio-skeletal syndrome with a mild defect in nucleotide excision repair and severe developmental failure. Am J Hum Genet. 2007;80:457–66. 17273966 10.1086/512486 PMC1821117

[13] Kashiyama K, Nakazawa Y, Pilz DT, Guo C, Shimada M, Sasaki K, et al. Malfunction of nuclease ERCC1-XPF results in diverse clinical manifestations and causes Cockayne syndrome, xeroderma pigmentosum, and Fanconi anemia. Am J Hum Genet. 2013;92:807–19. 23623389 10.1016/j.ajhg.2013.04.007 PMC3644632

[14] Imoto K, Boyle J, Oh K, Khan S, Ueda T, Nadem C, et al. Patients with defects in the interacting nucleotide excision repair proteins ERCC1 or XPF show xeroderma pigmentosum with late onset severe neurological degeneration. (Abstract). J Invest Derm. 2007;127:S92.

[15] Apelt K, White SM, Kim HS, Yeo JE, Kragten A, Wondergem AP, et al. ERCC1 mutations impede DNA damage repair and cause liver and kidney dysfunction in patients. J Exp Med*.* 2021;218:e20200622. 10.1084/jem.20200622 PMC7927433 33315086

[16] van den Heuvel D, Kim M, Wondergem AP, van der Meer PJ, Witkamp M, Lambregtse F, et al. A disease-associated XPA allele interferes with TFIIH binding and primarily affects transcription-coupled nucleotide excision repair. Proc Natl Acad Sci USA. 2023;120:e2208860120. 36893274 10.1073/pnas.2208860120 PMC10089173

[17] Tsodikov OV, Enzlin JH, Scharer OD, Ellenberger T. Crystal structure and DNA binding functions of ERCC1, a subunit of the DNA structure-specific endonuclease XPF-ERCC1. Proc Natl Acad Sci USA. 2005;102:11236–41. 16076955 10.1073/pnas.0504341102 PMC1183572

[18] Tsodikov OV, Ivanov D, Orelli B, Staresincic L, Shoshani I, Oberman R, et al. Structural basis for the recruitment of ERCC1-XPF to nucleotide excision repair complexes by XPA. Embo J. 2007;26:4768–76. 17948053 10.1038/sj.emboj.7601894 PMC2080803

[19] Orelli B, McClendon TB, Tsodikov OV, Ellenberger T, Niedernhofer LJ, Scharer OD. The XPA-binding domain of ERCC1 is required for nucleotide excision repair but not other DNA repair pathways. J Biol Chem. 2010;285:3705–12. 19940136 10.1074/jbc.M109.067538 PMC2823511

[20] Karczewski KJ, Francioli LC, Tiao G, Cummings BB, Alfoldi J, Wang Q, et al. The mutational constraint spectrum quantified from variation in 141,456 humans. Nature. 2020;581:434–43. 32461654 10.1038/s41586-020-2308-7 PMC7334197

[21] van der Meer PJ, Van Den Heuvel D, Luijsterburg MS, Unscheduled DNA synthesis at sites of local UV-induced DNA damage to quantify global genome nucleotide excision repair activity in human cells. Bio Protoc. 2023;13:e4609. 10.21769/BioProtoc.4609 PMC9909306 36816995

[22] Nakazawa Y, Yamashita S, Lehmann AR, Ogi T. A semi-automated non-radioactive system for measuring recovery of RNA synthesis and unscheduled DNA synthesis using ethynyluracil derivatives. DNA Repair. 2010;9:506–16. 20171149 10.1016/j.dnarep.2010.01.015

[23] Walden H, Deans AJ. The Fanconi anemia DNA repair pathway: structural and functional insights into a complex disorder. Annu Rev Biophys. 2014;43:257–78. 24773018 10.1146/annurev-biophys-051013-022737

[24] Wood RD. Mammalian nucleotide excision repair proteins and interstrand crosslink repair. Environ Mol Mutagen. 2010;51:520–6. 20658645 10.1002/em.20569 PMC3017513

[25] Abdullah UB, McGouran JF, Brolih S, Ptchelkine D, El-Sagheer AH, Brown T, et al. RPA activates the XPF-ERCC1 endonuclease to initiate processing of DNA interstrand crosslinks. Embo J. 2017;36:2047–60. 28607004 10.15252/embj.201796664 PMC5510000

[26] Masserot-Lureau C, Adoui N, Degos F, de Bazelaire C, Soulier J, Chevret S, et al. Incidence of liver abnormalities in Fanconi anemia patients. Am J Hematol. 2012;87:547–9. 22488129 10.1002/ajh.23153

[27] Cordts I, Onder D, Traschutz A, Kobeleva X, Karin I, Minnerop M, et al. Adult-onset neurodegeneration in nucleotide excision repair disorders (NERD(ND)): time to move beyond the skin. Mov Disord. 2022;37:1707–18. 35699229 10.1002/mds.29071

[28] Jones M, Beuron F, Borg A, Nans A, Earl CP, Briggs DC, et al. Cryo-EM structures of the XPF-ERCC1 endonuclease reveal how DNA-junction engagement disrupts an auto-inhibited conformation. Nat Commun. 2020;11:1120. 32111838 10.1038/s41467-020-14856-2 PMC7048804

[29] Mulderrig L, Garaycoechea JI. XPF-ERCC1 protects liver, kidney and blood homeostasis outside the canonical excision repair pathways. PLoS Genet. 2020;16:e1008555. 32271760 10.1371/journal.pgen.1008555 PMC7144963

[30] Weeda G, Donker I, de Wit J, Morreau H, Janssens R, Vissers CJ, et al. Disruption of mouse ERCC1 results in a novel repair syndrome with growth failure, nuclear abnormalities and senescence. Curr Biol. 1997;7:427–39. 9197240 10.1016/s0960-9822(06)00190-4

[31] McWhir J, Selfridge J, Harrison DJ, Squires S, Melton DW. Mice with DNA repair gene (ERCC-1) deficiency have elevated levels of p53, liver nuclear abnormalities and die before weaning. Nat Genet. 1993;5:217–24. 8275084 10.1038/ng1193-217

[32] Tian M, Shinkura R, Shinkura N, Alt FW. Growth retardation, early death, and DNA repair defects in mice deficient for the nucleotide excision repair enzyme XPF. Mol Cell Biol. 2004;24:1200–5. 14729965 10.1128/MCB.24.3.1200-1205.2004 PMC321450

[33] Gregg SQ, Gutierrez V, Robinson AR, Woodell T, Nakao A, Ross MA, et al. A mouse model of accelerated liver aging caused by a defect in DNA repair. Hepatology. 2012;55:609–21. 21953681 10.1002/hep.24713 PMC3250572

[34] Dolle ME, Kuiper RV, Roodbergen M, Robinson J, de Vlugt S, Wijnhoven SW et al. Broad segmental progeroid changes in short-lived Ercc1(-/Delta7) mice. Pathobiol Aging Age Relat Dis. 2011;1:7219. 10.3402/pba.v1i0.7219 PMC3417667 22953029

[35] Selfridge J, Hsia KT, Redhead NJ, Melton DW. Correction of liver dysfunction in DNA repair-deficient mice with an ERCC1 transgene. Nucleic Acids Res. 2001;29:4541–50. 11713303 10.1093/nar/29.22.4541 PMC92547

[36] Stern-Delfils A, Spitz MA, Durand M, Obringer C, Calmels N, Olagne J, et al. Renal disease in Cockayne syndrome. Eur J Med Genet. 2020;63:103612. 30630117 10.1016/j.ejmg.2019.01.002

[37] Schermer B, Bartels V, Frommolt P, Habermann B, Braun F, Schultze JL, et al. Transcriptional profiling reveals progeroid Ercc1(-/Delta) mice as a model system for glomerular aging. BMC Genomics. 2013;14:559. 23947592 10.1186/1471-2164-14-559 PMC3751413

[38] Hama EY, Nakamichi R, Hishikawa A, Kihara M, Abe T, Yoshimoto N, et al. Podocyte Ercc1 is indispensable for glomerular integrity. Biochem Biophys Res Commun. 2024;704:149713. 38428304 10.1016/j.bbrc.2024.149713

[39] Braun F, Mandel AM, Blomberg L, Wong MN, Chatzinikolaou G, Meyer DH, et al. Loss of genome maintenance is linked to mTORC1 signaling and accelerates podocyte damage. JCI Insight. 2025;10e172370. 10.1172/jci.insight.172370 PMC12220965 40392611

[40] Pontel LB, Rosado IV, Burgos-Barragan G, Garaycoechea JI, Yu R, Arends MJ, et al. Endogenous formaldehyde is a hematopoietic stem cell genotoxin and metabolic carcinogen. Mol Cell. 2015;60:177–88. 26412304 10.1016/j.molcel.2015.08.020 PMC4595711

[41] Mulderrig L, Garaycoechea JI, Tuong ZK, Millington CL, Dingler FA, Ferdinand JR, et al. Aldehyde-driven transcriptional stress triggers an anorexic DNA damage response. Nature. 2021;600:158–63. 34819667 10.1038/s41586-021-04133-7

[42] Niedernhofer LJ, Garinis GA, Raams A, Lalai AS, Robinson AR, Appeldoorn E, et al. A new progeroid syndrome reveals that genotoxic stress suppresses the somatotroph axis. Nature. 2006;444:1038–43. 17183314 10.1038/nature05456

[43] O’Neill AF, Meyers RL, Katzenstein HM, Geller JI, Tiao GM, Lopez-Terrada D, et al. Children’s Oncology Group’s 2023 blueprint for research: liver tumors. Pediatr Blood Cancer. 2023;70:e172370. 10.1002/pbc.30576 PMC10529117 37495540

[44] Schmid I, von Schweinitz D. Pediatric hepatocellular carcinoma: challenges and solutions. J Hepatocell Carcinoma. 2017;4:15–21. 28144610 10.2147/JHC.S94008 PMC5248979

[45] Schmid I, Haberle B, Albert MH, Corbacioglu S, Frohlich B, Graf N, et al. Sorafenib and cisplatin/doxorubicin (PLADO) in pediatric hepatocellular carcinoma. Pediatr Blood Cancer. 2012;58:539–44. 21922643 10.1002/pbc.23295

