# Supplementary material for: A new multisystem ERCC1-hepatorenal syndrome: insights from a clinical cohort, molecular pathogenesis, and management guidelines
Source: Eur J Hum Genet. 2025 Jul 19;33(10):1252–63. doi: 10.1038/s41431-025-01910-0 (PMC12480883; doi:10.1038/s41431-025-01910-0)
Supplement: Supplementary file 1 — supplemental information [file 41431_2025_1910_MOESM1_ESM.docx]

**Supplemental information**

Case reports

*Individual PV50LD*

Individual PV50LD is 18 years of age, the eldest of three siblings to healthy, unrelated parents of mixed ethnicity including Indigenous Australian, Maltese, and Anglo-Celtic heritage. During the pregnancy, her mother had a transient dilated cardiomyopathy that resolved postpartum. She was born at 39 weeks gestation, and her birth weight was 1.9 kg (Z = −3.8), length 44 cm (Z = −2.44), and head circumference 29.5 cm (Z =−3.8). She had poor growth in infancy, and at 18 months of age, she was noted to have liver dysfunction with a predominantly cholestatic pattern. Liver ultrasound and magnetic resonance cholangiopancreatography were normal. Liver function progressively declined, as evident from progressive increases in γ-glutamyltransferase (GGT), alanine transaminase (ALT), and bilirubin levels. At a liver biopsy (at age 3.5 years), the lobular parenchyma showed variation in hepatocyte nuclear morphology, with some much larger nuclei and cells with double nuclei to areas where the cells have small, unremarkable nuclei. There was mild portal fibrosis and mild fibrous portal expansion and mild focal interface inflammation with no ductopenia or periductal fibrosis.

She experienced recurrent infections including tonsillitis, chicken pox, hand-foot-and-mouth disease, pneumonia, bronchitis, and recurrent episodes of fever, abdominal pain, and pale stools with no cause found. Immunological investigations did not identify any immunodeficiency. She developed episodes of ocular and skin photosensitivity. At age 6 years, renal dysfunction was detected, with features suggestive of proximal tubular dysfunction characterized by albuminuria (sub-nephrotic range) and hypercalciuria. Her renal function fluctuated, with intermittent episodes of acute kidney injury, progressive kidney impairment with increasing creatinine levels, and minimal response to acetyl cholinesterase inhibition. Renal ultrasound showed small kidneys with increased echogenicity and reduced corticomedullary differentiation. Following liver transplant, her tubulopathy stabilized, and then followed a fluctuating course of frequent acute kidney injury with worsening baseline function over time. At age 18 years, she developed stage 5 chronic kidney disease and was under consideration for a kidney transplant.

Lung function tests showed moderate restrictive lung disease. Developmental milestones were normal, but some learning difficulties were evident at school age. Cognitive assessment at age 16 showed a mild intellectual disability (IQ 68). Vision and hearing were normal. Growth remained very slow despite supplemental feeding. She developed progressive liver impairment and underwent orthotopic liver transplantation at age 9 year 10 months. At age 12 years, ovarian insufficiency was diagnosed. Brain magnetic resonance imaging (MRI) at age 12 years showed mild cerebral atrophy with moderate cerebellar atrophy and mild brainstem atrophy.

Exocrine and endocrine pancreatic insufficiency were diagnosed at age 14 years and she began insulin treatment and Creon. She developed hypothyroidism and commenced thyroxine replacement aged 16 years. She developed features of a cerebellar ataxia with a wide-based stance, dysmetria, tremor and poor co-ordination. Mobility is limited by a combination of ataxia and fatigue, and she requires a wheelchair for more than short distances.

Despite rigorous sun protection, she has had a total of six basal cell carcinomas removed from her face, from the age of 15 years.

At last assessment aged 18 years, her height was 139cm (Z-3.72) and weight 28kg (Z-8.8). She had a slim build, limited muscle bulk and subcutaneous fact. She had deep-set eyes and micrognathia. She had freckling in sun-exposed areas. She had a mild kyphosis, fifth finger clinodactyly, narrow feet and short fourth and fifth toes.

*Individual PV46LD*

Individual PV46LD is 15 years and 10 months of age, a younger sister to individual PV50LD. The pregnancy was complicated by placenta previa and transient maternal cardiomyopathy, and she was born at 35 weeks’ gestation with a birth weight of 1.79 kg (third centile), a length of 45 cm (50th centile), and a head circumference of 29 cm (less than third centile). She exhibited failure to thrive in the first year of life and liver impairment from age 2 years, with significantly increased GGT, ALT, and bilirubin levels. Liver biopsy at age 6 years showed damage to the intrahepatic bile ducts, resulting in periductal fibrosis. As with the liver biopsy from her sister, a moderate number of double-nucleated hepatocytes was seen, some with large nuclei and large nucleoli.

Individual PV46LD developed tubular dysfunction by six years, which fluctuated over time but with progressive renal impairment and impaired creatinine clearance. Renal ultrasound showed small kidneys with reduced corticomedullary differentiation and nephrocalcinosis. She had episodes of ocular and skin photosensitivity and early recurrent infections. Developmental milestones were normal, but a mild intellectual disability (IQ 63) was diagnosed at school age. There has been slow forward progress with neurodevelopmental milestones. Vision and hearing were normal. Liver impairment was progressive, and at age 8 years, she underwent liver transplantation. Brain MRI at age 5 years was normal, but a repeat MRI at 10 years showed moderate cerebellar atrophy and mild cerebral atrophy. At age 11 years, she had a basal cell carcinoma removed from her scalp, with vigilant sun protection instituted years prior. She had ovarian insufficiency diagnosed. Hypothyroidism was diagnosed and treated with thyroxine. She has exocrine pancreatic insufficiency treated with Creon but no diabetes.

In her teenage years she developed a wide-based stance, dysmetria and poor coordination. She has limited mobility due to fatigue and ataxia and uses a wheelchair for mobility for more than short distances.

At last assessment at age 15 years 10m, growth was slow with height of 135cm (Z-4.23) and weight of 24kg (Z-10). She had deep-set eyes, medial extension of the eyebrows with the appearance of synophrys, retrognathia and fifth finger clinodactyly. She had a wide-based gait and a tremor. She had freckling of sun-exposed areas. She had progressed to stage 5 chronic kidney disease and was under consideration for kidney transplant.

*Individual CA16LD*

Individual CA16LD was born at term via Cesarean section due to fetal distress. There was an isolated echogenic abdominal focus noted at the second trimester anatomy scan, but otherwise the pregnancy was uncomplicated. His birth weight was 2188 (1^st^ percentile), length was 45.5cm (5^th^ percentile) and head circumference was 31.3cm (2^nd^ percentile). He did not require significant resuscitation or NICU admission, but in the weeks following delivery he had persistent feed intolerance and poor growth. This improved somewhat with the use of a hydrolyzed formula and prescription of a protein pump inhibitor. General blood chemistries revealed neonatal cholestasis, with slight elevations in bilirubin, GGT and ALP that resolved with time. Abdominal ultrasonography showed nephrocalcinosis. Screening biochemical investigations showed a significant elevation in methionine (0.931 mmol/L) and total homocysteine (40.3umol/L). A methionine metabolism disorder was initially suspected and a methionine restricted diet was initiated at 3 months of age, which resulted in rapid normalization. The diet was shortly discontinued and the normalization of both amino acids in the plasma was persistent. Panel testing revealed no abnormalities in any known genes associated with methionine demethylation. Subsequently, trio exome sequencing identified the causal homozygous variant in *ERCC1* at 7 months of age. The family is of non-consanguineous mixed Western European ancestry.

Now 3 years old, individual CA16LD remains very small for age, with a length of 89.6 cm (−2.6 SD), a weight of 11.6 kg (−3.8 SD), and a head circumference of 47.2 cm (5th percentile). Feeding has been complicated by significant oral aversion, though this has improved with advancing age and he remains orally fed. He has remarkable skin and ocular photosensitivity, with a history of severe skin reactions to even limited sun exposure. There is a delay in expressive speech with only 3 words at 2 years and no 2-word combinations, though he can understand 2-step commands and shows normal gross and fine motor development . On examination he is non-dysmorphic but has short appearing palpebral fissures and widespread café-au-lait macules over his body, which were not present in early infancy. There are no noted congenital anomalies, though skeletal radiographs have never been pursued.

He has normal hepatic function, but there are persistent, mild elevations of ALT, AST and GGT. The first AFP measured at 2 years and 8 months was 83 ug/L (ref <9 ug/L). His AFP then acutely rose to 269 ug/L on routine screening approximately 7 months later. The AFP continued to rise was 1076 ug/L at 3 years 11 months of age (time of writing). At 2 years of age, his baseline US identified a small hemangioma. In the context of the rising AFP and concern for HCC, a contrast enhanced US was done and identified 2 additional liver lesions, one of which had features concerning for HCC. A follow up abdominal MRI completed within 2 weeks could not definitely characterize the lesion as HCC given its small size (<1cm). However, biopsy of the lesion showed features consistent with small early or small progressive HCC. The patient is currently undergoing liver transplant evaluation. His renal function is also normal. MRI brain is normal. Audiologic evaluation at 11 months of age was normal. Ophthalmologic assessment at 1 year of age showed blonde fundi and impaired visual acuity. Chromosomal breakage studies with diepoxybutane and Mitomycin C showed increased rates of chromosome breakage. There was an 8-fold higher level of abnormal breakage and triradial/multiradial structures compared to controls, while individuals with chromosome instability syndromes typically show a 10-fold higher level of breakage. He has a normal full blood examination. While he does not show any other unique features of Fanconi anemia, we hypothesize that the café-au-lait macules may be a manifestation of impaired interstrand cross-linked repair.

*Individual XE28CH*

Individual XE28CH first presented to genetics clinic at 9 years of age. She is the eldest of two siblings to healthy, unrelated parents of European descent. Her mother had pre-eclampsia toward the end of the pregnancy, but the pregnancy was otherwise uncomplicated. She was born full-term and her birth weight was 2.55 kg. She had a history of poor growth throughout infancy and childhood, with weight continuously below 1^st^ percentile despite nutritional supplementation. She was frequently hospitalized in her first two years of life due to recurrent urinary tract infections. Developmental milestones were reported to be normal. She had a history of myopia and amblyopia. Hearing was normal. She had a very slim build with poor muscle bulk and a paucity of subcutaneous fat. She had developed freckling on sun-exposed areas. She had mildly deep-set eyes, and her scalp hair was thin.

She was seen by an endocrinologist, who did a growth hormone challenge test and recommended growth hormone replacement therapy, but this treatment option was never pursued. At age 7 years, she began to develop increasing numbers of telangiectasias on her arms and legs. She experienced ocular and skin photosensitivity. At age 9, she was referred to hepatology due to elevated transaminases. Liver ultrasound at the time showed heterogenous echogenic liver without lesions. Abdominal MRI revealed a cirrhotic liver with numerous regenerative biliary nodules in areas of fibrosis. Liver biopsy was suggestive of biliary pattern advanced fibrosis. Liver copper content was elevated, suggesting a possible diagnosis of Wilson’s disease. Follow-up testing revealed elevated 24-hour urine copper (62 ug/L; reference range 3-35) and elevated copper/CRT ratio (182 ug/L; reference range 0-49). She was started on chelation therapy with penicillamine. Eye examination showed myopia but was negative for Kayser Fleischer rings and sunflower cataract. Genetic testing via a copper metabolism disorders panel was negative for pathogenic variants in ATP7B and ATPB7 peptide analysis was normal, both refuting the possible diagnosis of Wilson’s disease. Chelation therapy was discontinued.

Evaluation by nephrology revealed glucosuria, proteinuria, hyperphosphaturia, metabolic acidosis, and hypophosphatemia – suggestive of Fanconi renotubular phenotype.

Chromosomal microarray showed a normal female karyotype. Trio exome sequencing revealed biallelic variants in the *ERCC1* gene (maternally inherited pathogenic p.Arg156Trp variant; paternally inherited likely pathogenic canonical splice site variant, IVS6-2A>G). Mitochondrial genome analysis was negative. Chromosomal breakage studies with diepoxybutane and Mitomycin C showed increased rates of chromosome breakage. Laboratory results revealed progressively rising AST, ALT, GGT, bilirubin, and AFP levels and liver transplant was discussed. Abdominal MRI obtained at that time revealed progression of cirrhosis with multinodular morphology and three new liver nodes, which were determined to be hepatocellular carcinoma on biopsy. PET scan indicated that the HCC had spread to her right lung.

She was not a candidate for liver transplantation due to metastatic disease. Chemotherapy options were limited due to her underlying DNA repair disorder. At the time of her last evaluation (aged 11.75 years), she was 129 cm in height (z-score -2.75) and weighed 20.8 kg (z-score -4.38). Laboratory values showed significant hepatic and renal dysfunction: AFP of 30,047 ng/mL, ALT of 102 IU/L , AST of 338 IU/L, Total bilirubin of 23.8 mg/dL, direct bilirubin of 15.0 mg/dL, creatinine of 1.43 mg/dL. Individual XE28CH died at the age of 11 years due to complications of metastatic hepatocellular carcinoma.

*Individual XE23CI*

Individual XE23CI presented at the age of 8 years and 3 months with new onset jaundice and a one month complaint of abdominal pain. Prior to this presentation, he was known for microcephaly, poor weight gain and, developmental delay, ADHD. In school, there were some concerns about his learning and cognitive skills and he performed a few years behind what was expected, kindergarten level in 3^rd^ grade. He developed tics at age 7, but no additional evaluation was done at that time. He was otherwise in good health. The participant was also noted to be mildly dysmorphic with a high narrow nasal bridge, deeply set eyes, short palpebral fissures (not noted but visible on photos and discussed at that time), and triangular face. He had increased malar freckling.

MRI showed evidence of a cirrhotic liver with multiple nodules, highly suspicious for hepatocellular carcinoma. The dominant lesion was centered within the left hepatic lobe, measured approximately 4.6 x 4.4 x 4.1 cm and demonstrated heterogenous arterial phase enhancement as well as washout during the portal venous phase. The alphafetoprotein (AFP) level was greater than 90,000 ng/ml. Guided biopsy of the lesion confirmed hepatocellular carcinoma of the macrotrabecular subtype that was well differentiated. The background liver showed chronic hepatitis and borderline paucity of intralobular bile ducts. A pulmonary scan confirmed no distant metastasis.

It was decided to treat him with chemotherapy as per the AHEP1531 group F and he received cisplatin and doxorubicin. Within a few days of receiving chemotherapy, his transaminases started to rise and he developed coagulopathy and encephalopathy. He also experienced profound bilateral hearing loss, when his baseline impairment was only mild to moderate. He was rapidly evaluated for liver transplantation and was listed as a candidate.

The clinical findings with microcephaly and small size were noted to overlap with chromosome breakage disorders Fanconi anemia, Bloom syndrome, and Warsaw breakage syndrome as differential diagnoses considered, and whole exome sequencing was initiated. Two weeks after he received the first cycle of chemotherapy, rapid whole exome sequencing revealed two pathogenic compound heterozygous variants in *ERCC1*. This result did not change the decision to list him for transplantation.

His course was complicated by KDIGO stage 3 acute kidney injury, thought to be consistent with nephrotoxicity secondary to cisplatin although more severe than expected. It was decided to place a hemodialysis catheter to treat fluid overload and azotemia. During this procedure, a kidney biopsy was performed and revealed global kidney injury without evidence of glomerulonephritis. A bone marrow biopsy was also done and showed normocellular marrow for age with trilineage hematopoiesis and myeloid and megakaryocytic hypoplasia as well as stromal injury with histiocytic hyperplasia. The bone marrow aspirate confirmed no diagnostic immunophenotypic abnormalities by flow cytometry and no numeric or structural chromosomal abnormalities detected by FISH.

The participant’s liver dysfunction progressed and he developed profound cholestasis. He had fluctuations in his mental status and a three day course of Molecular Adsorbent Recirculating System (MARS) therapy was attempted followed by a trial of N-acetyl cysteine. However, six weeks after initial presentation, his condition significantly deteriorated as he developed gastrointestinal bleeding, pneumococcal bacteremia, and cardiorespiratory failure requiring vasopressors and invasive mechanical ventilation. After discussions between the medical teams involved and the family, active care was withdrawn and the participant passed away.

*Individual XE24CI*

Individual XE24CI is the brother of individual XE23CI. He had a history of failure to thrive, global developmental delay and severe photosensitivity. At the age of 6 months, he experienced a severe blistering sunburn after 10 minutes of sun exposure and required hospitalization. He was also known for microcephaly and dysmorphic features that included a triangular-shaped face and deeply-set eyes. He had skin findings of multiple café-au-lait macules and hypo-pigmented lesions.

He was seen by our team for the first time at 5 years of age when his brother was diagnosed with an *ERCC1*-related DNA repair defect. Exome sequencing confirmed that individual XE24CI harbored the same biallelic variants. Because of his brother’s history, a surveillance plan that included namely serial MRIs and serum AFP was put into place for him.

At the start of his follow-up, he had mild elevation of transaminases and GGT and was started on ursodeoxycholic acid for its choleretic and cytoprotective benefits. We trialed a “mitochondrial cocktail" (that included Coenzyme Q10, carnitine, vitamins C/B1/B6/E) to mitigate reactive oxygen species-mediated cellular injury. However, he had difficulty swallowing the medications and these were discontinued.

During follow-up, individual XE24CI was evaluated by neurology and confirmed to have motor tics, gait ataxia, and mild peripheral hypotonia. His audiology testing showed evidence of borderline normal hearing in the right ear with a slight sensorineural component at 4000 Hz only. He has an intellectual disability and learning in school seems to have become progressively more challenging.

On surveillance imaging, MRI with contrast, the liver parenchyma appeared fibrotic with nodularity likely reflecting dysplastic or regenerative nodules. In a timeframe of three months however, a new hepatic lesion near the junction of segments 5 and 4B measuring 0.9 x 0.7 appeared and had characteristics concerning for malignancy. Concomitantly, AFP levels rose from a normal range to a peak of 459.4 ng/ml. The lesion was biopsied and confirmed to be moderately differentiated hepatocellular carcinoma with a background liver that showed evidence of significant bridging fibrosis and regenerative nodule formation. After being biopsied, radiofrequency ablation was performed on the lesion. Because of the multifocal lesions, there was a discussion about offering locoregional treatment versus undergoing an evaluation for liver transplantation which the family opted for. Leading up to liver transplantation, the patient struggled with poor appetite and achieving an optimal nutritional status was challenging.

The patient underwent liver transplantation at the age of 10 years and 11 months. Before transplantation, he had evidence of mild renal function impairment with a GFR of 79 ml/min. To minimize nephrotoxicity, his immunosuppression induction regimen consisted of basiliximab, corticosteroids and tacrolimus. His maintenance regimen has been a combination of sirolimus and tacrolimus. At the last assessment, he was 8 months out of transplantation and his course has been overall unremarkable. However, he did develop hypertension and insulin dependent diabetes. As he was experiencing intermittent vomiting, dysphagia and weight loss, he underwent an esophagogastroduodenoscopy that revealed candida esophagitis for which he was treated. He was also started on overnight feeds and has had better weight gain since. He experienced an episode of acute cellular rejection at 16 months after liver transplant shortly after transition from tacrolimus to sirolimus based immunosuppression. He requires ongoing treatment of diabetes mellitus with insulin which is unusual for transplant recipients in this age group and may point to *ERCC1*- associated pancreatic dysfunction. The patient is alive and well at 18 months after the liver transplant.

*Individual XE1AH*

Individual XE1AH first presented to genetics clinic at 16 years of age. The primary reason for referral was severe and progressive liver disease. She is the youngest of two siblings to healthy, unrelated parents of European descent. The pregnancy was complicated by intrauterine growth retardation, and due to this, birth was induced at GA 36 weeks (birth weight 1.735kg (-3SD)). She had a history of poor growth throughout infancy and childhood. Developmental milestones as well as hearing and vision were reported to be normal. She experienced ocular and skin photosensitivity, reported missing permanent teeth and recurrent lower airway infections; FEV1 and FVC were decreased with negative reversibility test and further evaluation is pending. On examination, she had a very slim build with poor muscle bulk and a paucity of subcutaneous fat. She had petite facial features with a small face and a small, pointed nose, and bilateral hypoplasia of the thenar eminence and reduced flexion of both thumbs, as well as gracile, tapering fingers: radiographs of hands were normal. She had developed freckling on sun-exposed areas, and had several café-au-lait macules (two on the back and one on the dorsum of the hand). At age 14 years, she had been referred to the local pediatric gastroenterology unit due to slightly elevated ALT of 57 U/L measured as part of screening due to poor weight gain. Apart from the low growth, she was feeling well. Liver ultrasound at the time was normal. Eventually, she was referred to a tertiary pediatric hepatology unit due to unexplained progressive elevation of liver enzymes. At the age of 17 years, her ALT was 213U/L, GGT 619 U/L, bilirubin 35µmol/L, ALP 661 U/L, and INR 1.2. Now liver ultrasound showed irregular echogenicity and fibrosis with signs of cirrhosis. The bile ducts were normal. MRCP further revealed multiple hypointense focal liver lesions, possible dysplastic nodules, and a 7mm long stenosis of the common hepatic duct. The Fibroscan measurement was 32kPa indicative of cirrhosis. Liver biopsy showed inactive cryptogenic cirrhosis with bile duct proliferation, mild inflammatory changes and slight cholestatic changes.

She had been started on ursodeoxycholicacid at age 16 years, but despite this her liver function continued to deteriorate. Gastroscopy was performed due to falling platelets and signs of cirrhosis. This showed three small grade 1 esophageal varices. AFP was 8*10^3 IE/ml (borderline elevated). Ultrasound of kidneys was normal. BP and creatinine were normal, but due to low free carnitine and glucosuria, we suspect proximal tubular dysfunction, and further kidney evaluation is pending. During hepatological workup, the patient was diagnosed with primary ovarian insufficiency at age 16 years. MR scan showed atrophic ovaries and a normal but hypotrophic uterus. Genetic testing via a liver disorders panel revealed a heterozygous known pathogenic variant in *DHCR7* representing carrier state for Smith-Lemli-Opitz syndrome. 7-dehydrocholesterol levels were in accordance with this. Trio-genome analysis revealed homozygosity for a pathogenic variant in the *ERCC1* gene (c.796G>C).

**Supplemental Methods – Genomic sequencing**

Individuals PV50LD and PV46LD underwent quad whole genome sequencing as previously published^1^. Individual CA16LD underwent trio exome sequencing at Blueprint Genetics, Helsinki.

Individual XE28CH underwent trio exome sequencing performed by GeneDx (Gaithersburg, MD, USA). Genomic DNA from both individuals XE23CI and XE24CI was sequenced by GeneDx (Gaithersburg, MD, USA)

*XE23CI*

Using genomic DNA from the submitted specimen, the exonic regions and flanking splice junctions of the genome were captures using a proprietary system developed by GeneDx and sequenced by massively parallel (NextGen) sequencing on an Illumina system with 100bp or greater pair-end reads. Reads were aligned to reference gene sequence based on human genome build GRCh37/UCSC hg19 and analysed for sequence variants using a custom-developed analysis tool (Xome Analyzer). Reported clinically significant variants were confirmed by an appropriate orthogonal method in the proband and relevant relatives. Sequence alterations were reported according to Human Genome Variation Society (HGVS) nomenclature guidelines.

*XE24CI*

Using genomic DNA from the submitted specimen, the relevant portion of the requested gene was PCR amplified and capillary sequencing was performed. Bi-directional sequence was assembled, aligned to reference gene sequence based on human genome build GRCh37/UCSC hg19 and analysed for known familial variants. Sequence alterations were reported according to Human Genome Variation Society (HGVS) nomenclature guidelines. The methods used by GeneDx are expected to be greater than 99% sensitive in detecting variants identifiable by sequencing.

*XE1AH*

Individual XE1AH underwent Whole Genome Sequencing (WGS) performed using DNA from blood from the girl and both parents. DNA was prepared with Illumina DNA PCR Free Library Prep and sequenced with NovaSeq (Illumina) at the NGC WGS-West Core facility at Aarhus University Hospital, Denmark. Average coverage was 30x. Data analysis was performed using NGC bioinformatics analysis (NBA2) and subsequently using VarSeq. Data was filtered for autosomal dominant *de novo* variants and variants consistent with autosomal recessive inheritance.

**
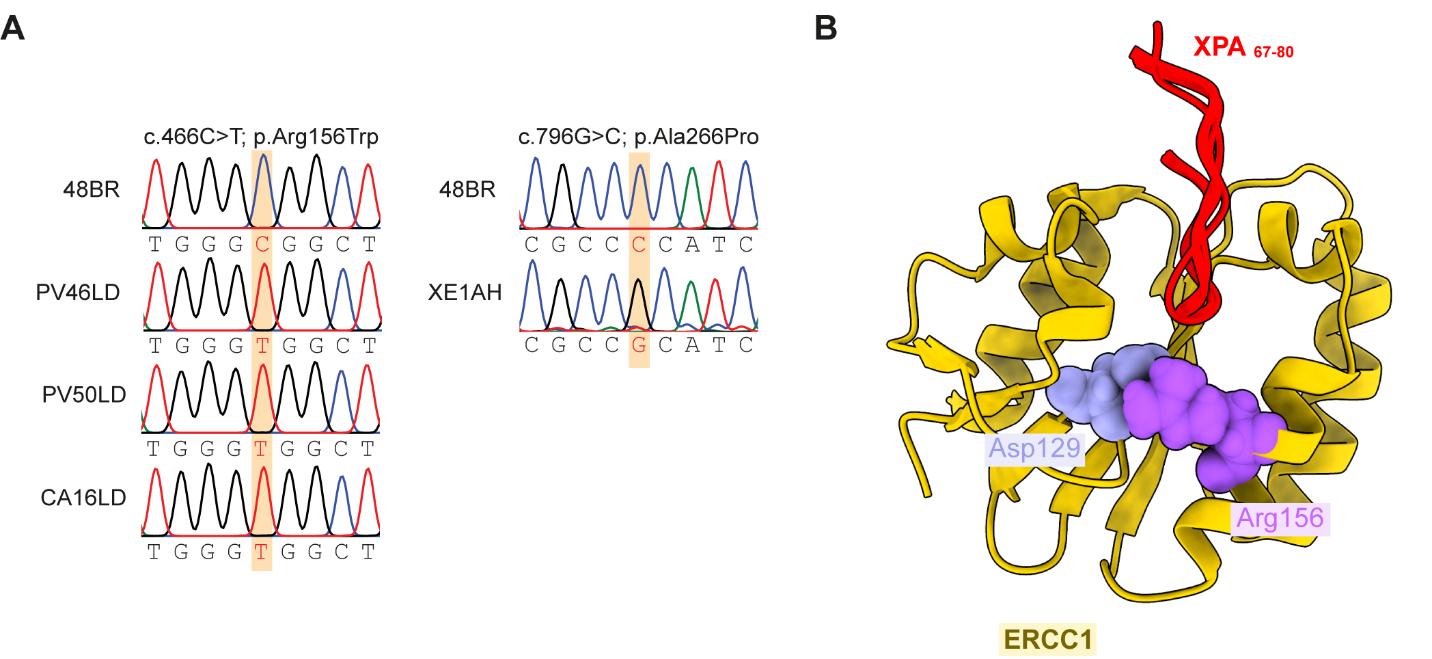
**

**Figure S1.** (A) Sanger sequencing of gDNA from 48BR (WT), PV46LD, PV50LD, CA16LD, and XE1AH. We sequenced a region of the ERCC1 gene that includes the missense variant. (B) Solution NMR structure of the ERCC1 central domain (yellow) bound to a short XPA peptide (red), based on 2JNW. Residues that form a salt bridge just below the XPA-binding pocket (Arg156, Asp129) are indicated. The Arg156 residue is substituted for Trp in all individuals in our cohort, except for XE1AH who carries the p.Ala266Pro variant. Related to Figure 1B, C.

**Structural Insights on ERCC1 patient variants**


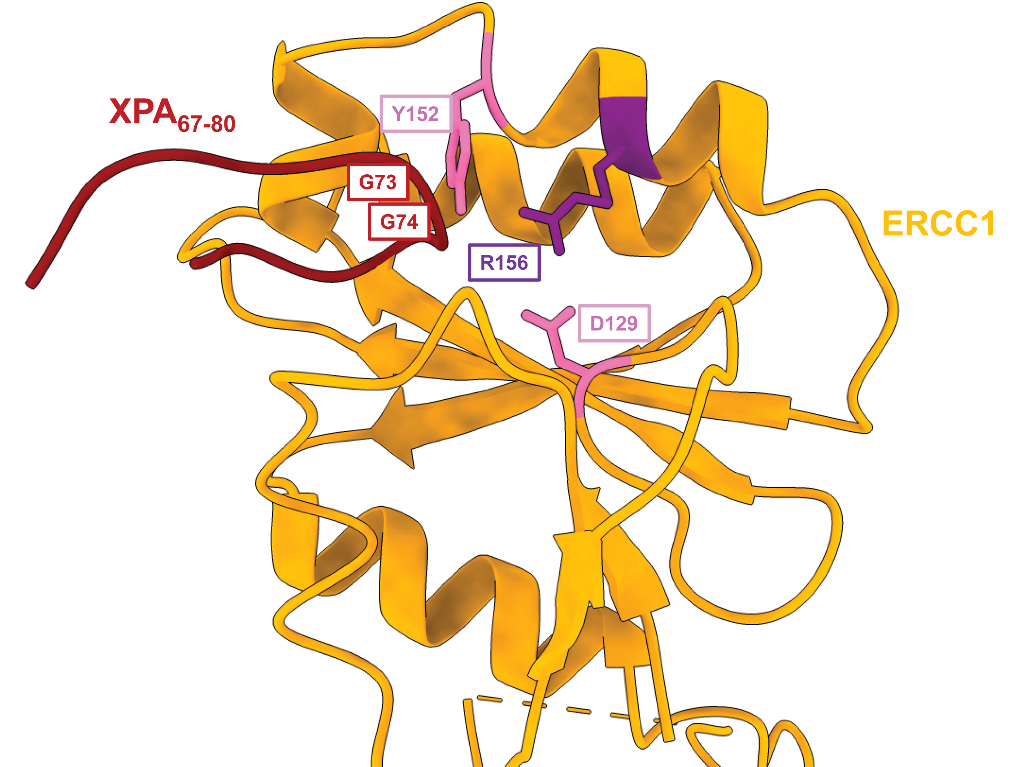
***Arg156Trp***Arg156 is situated in the middle domain of ERCC1 near the XPA-binding pocket and it appears to play a central role in stabilizing the structure of the domain. Its side chain forms a hydrogen bond with the side chain hydroxyl group of Tyr152, a structural element that stabilizes this region of the helix. Importantly, the Tyr152 side chain directly contacts the main chain amides of XPA glycine residues Gly73 and Gly74. In addition, as mentioned in the previous study, a salt bridge is formed between the side chains of ERCC1 Asp129 and Arg156. Substituting Arg156 for Trp is predicted to disrupt the hydrogen bond with Tyr152 and correspondingly, the packing of the Y152 side chain. Changes in the positioning of this side chain are expected to alter its contacts with XPA Gly73 and Gly74 and therefore, the recruitment of ERCC1-XPF to the site of damage in NER and XPA’s orientation within the larger NER complex. Substitution of Arg156 with Trp will also disrupt the salt bridge with Asp129, which is expected to alter and destabilize the structure. The addition of the heteroaromatic side chain of Trp has the potential to reorganize the packing of the hydrophobic core of the domain, for example, packing the side chain down into the core of the domain, pulling the helix ‘down’ in the orientation of the figure below. Overall, the R156W substitution most likely affects the structure of the XPA-binding pocket and the recruitment of ERCC1-XPF into NER machinery. However, because it is not directly in the binding interface, R156W might have a less severe effect on NER compared to N110/Y145A variant in the ERCC1-XPA interface.­­­­­­­­­­­­­­

**Figure S2.** Structure of the ERCC1-XPA complex with Arg156 highlighted in magenta (PDB:2JNW). Arg156 is a central residue in this part of the domain forming key interactions with neighboring residues: Asp129 and Tyr152.


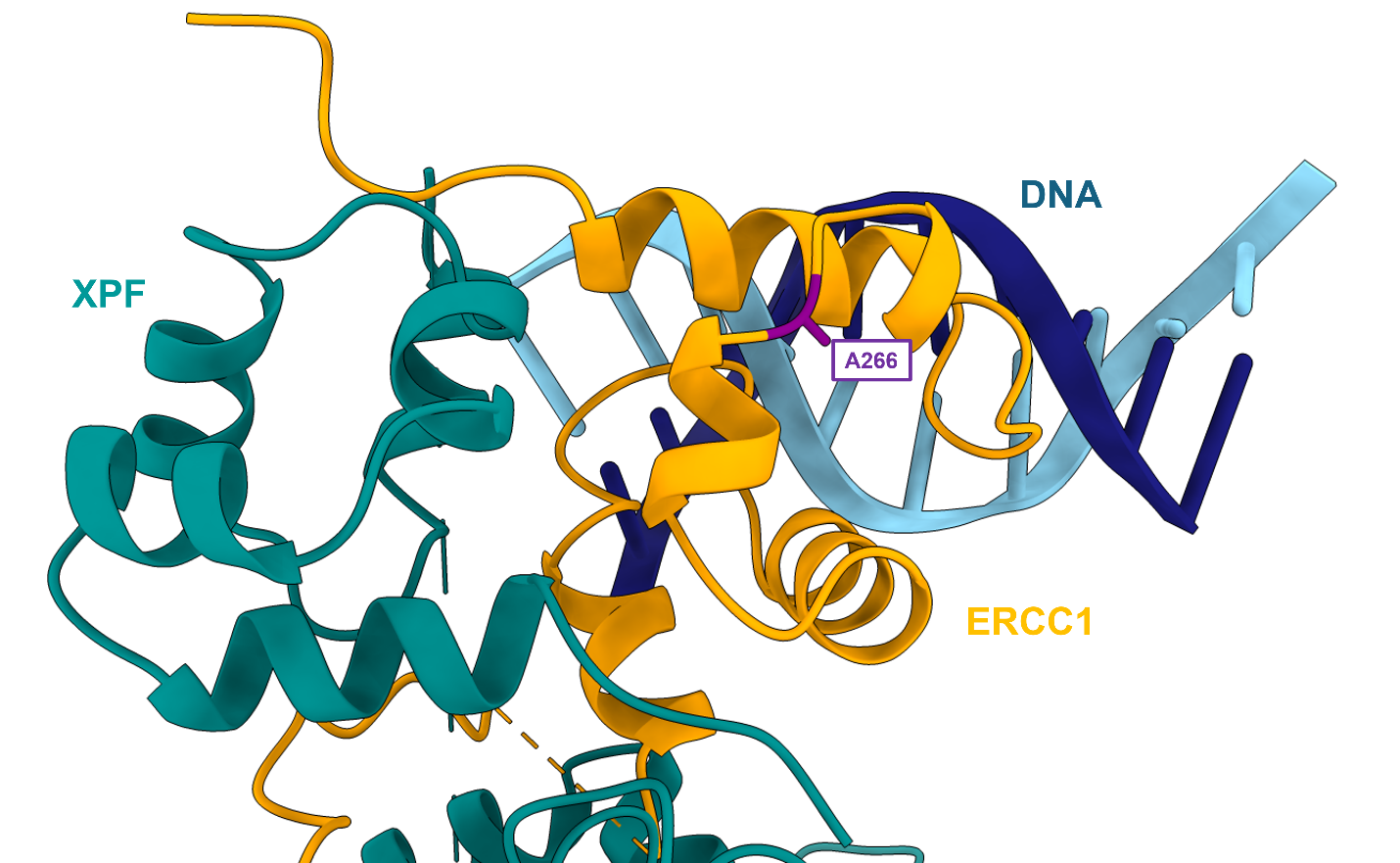
***Ala266Pro***ERCC1 Ala266 is located at a major kink in the structure of the (HhH)_2_ domain between the XPF interacting region and the C-terminal helical motif that interacts with dsDNA. Cryo-EM structures of the DNA-free and DNA-bound ERCC1-XPF heterodimer suggest that the ERCC1 (HhH)_2_ domain and the XPF (HhH)_2_ domain need to be substantially reorganized to switch from the autoinhibitory configuration to engage dsDNA and promote incision. Substitution of Ala266 to Pro is predicted to affect the conformation and dynamics of this segment of the protein. The stereochemical restraints of proline are expected to inhibit the requisite dynamic rearrangements within the ERCC1 (HhH)_2_ domain required for DNA binding as well as its contacts with the XPF (HhH)_2_ domain.

**Figure S3.** Cryo-EM structure of ERCC1 in complex with the XPF (HhH)2 domain and DNA (PDB:6SXB). Residue Ala266 at the major kink in the structure is colored magenta.


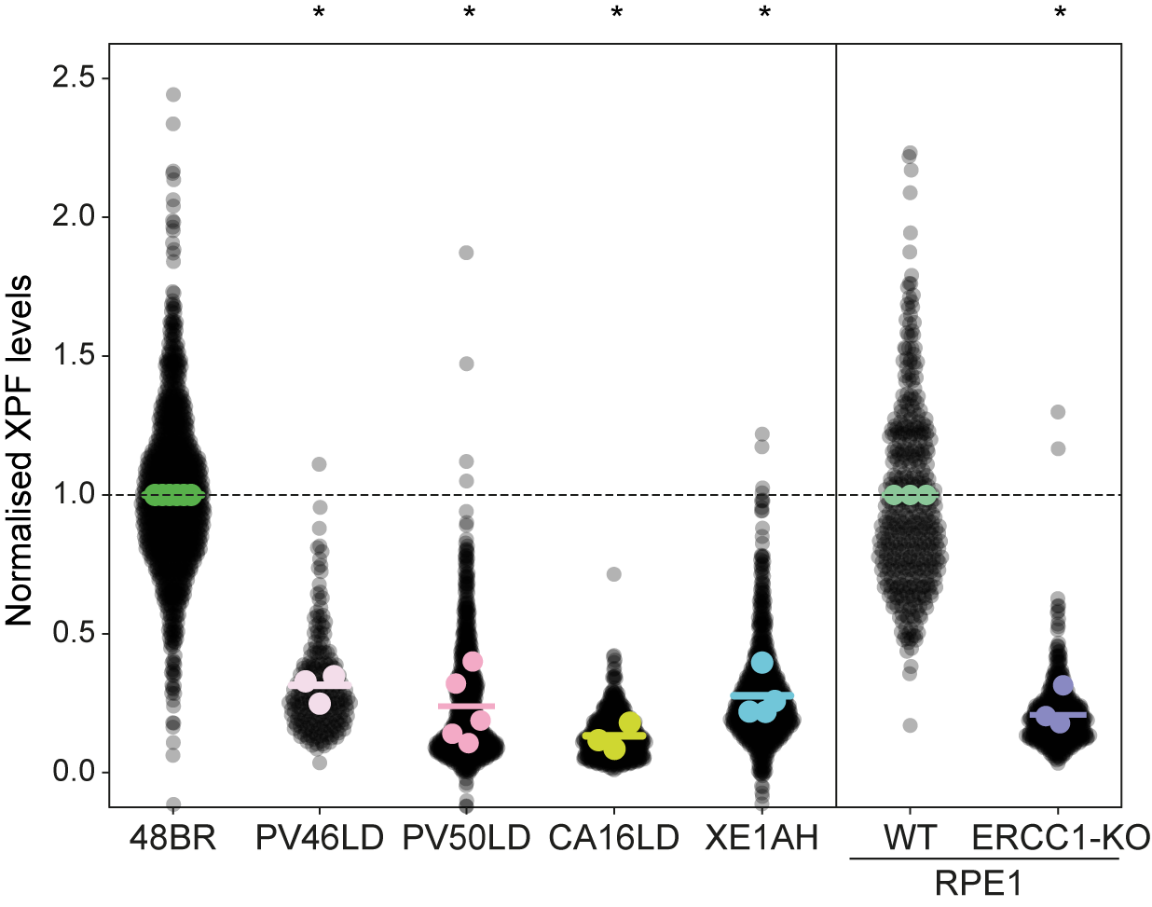


**Figure S4. XPF levels are reduced in patient cells.** Quantification of the intensity of immunofluorescently labelled ERCC1 in the nuclei (stained with DAPI) of PV46LD, PV50LD, and CA16LD cells, as well as in wildtype (48BR and RPE) and ERCC1-KO. Protein levels in patient fibroblasts were normalised to control fibroblasts 48BR, and levels in RPE ERCC1-KO were normalised to those in RPE WT. Each datapoint represents one cell, with the colored bar depicting the mean of all datapoints, and the colored points representing the mean of the individual replicates. The scale bar is 10 µm. Statistical significance was determined by a paired 2-tailed t-test (* = p<0.05). Related to Figure 2B, C.

**
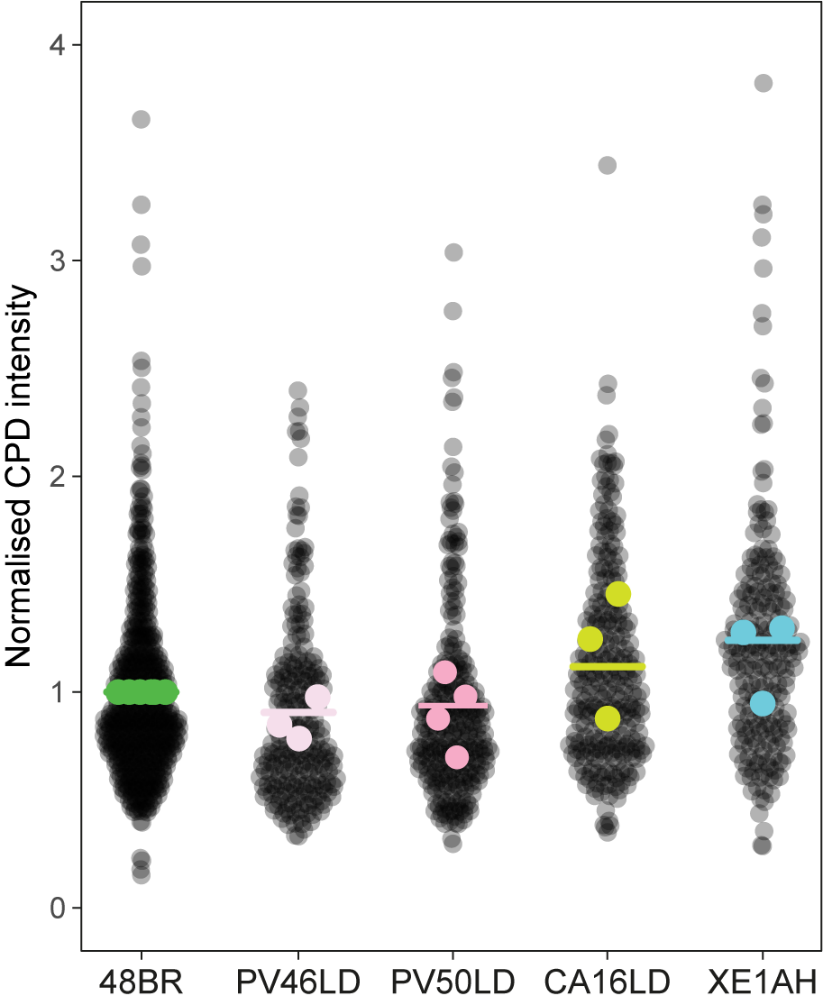
**

**Figure S5. Contols for UDS experiments.** Quantification of the CPD intensity at the site of local damage in ERCC1 patient fibroblasts at 1 h after 30 J/m² local UV irradiation. Related to Figure 3A, B. The CPD intensity is normalized to wildtype 48BR. Each datapoint represents one local damage site, with the colored bar depicting the mean of all datapoints.

**Table S1: Cell lines**

| Cell line | Genotype | Origin |
| --- | --- | --- |
| 48BR | Wild-type | Alan Lehmann (University of Sussex, UK) |
| CA16LD | *ERCC1*  Allele 1+2: c.466G>A; p.Arg156Trp | Alexandra Carter, Rebecca Sparkes and Eliza Phillips (University of Calgary, Canada) |
| PV46LD | *ERCC1*  Allele 1: c.466G>A; p.Arg156Trp  Allele 2: p.142-175Δ | Apelt et al., 2021^1^ |
| PV50LD | *ERCC1*  Allele 1: c.466G>A; p.Arg156Trp  Allele 2: p.142-175Δ | Apelt et al., 2021^1^ |
| XE1AH | *ERCC1*  Allele 1+2: c.790G>C; p.Arg266Trp | Pernille Axél Gregersen (Aarhus University Hospital, Denmark) |
| RPE1 ERCC1-KO (cl. 16) | ERCC1 knockout in RPE1 | Apelt et al,.2021^1^ |
| RPE1-hTERT-iCas9-PuroS-TP53-KO (abbreviated to RPE1 WT) | RPE1-hTERT cells expressing inducible Cas9 (iCas9) knockout for TP53 and PuroS | Rob Wolthuis lab^2^, Amsterdam UMC |

**Table S2: Primers**

| Primers |  | Sequence |  |
| --- | --- | --- | --- |
| Missense PCR Sanger Sequencing | Fw-seq | CTGGTCTTCTAGGTAAGCACAG | oML #466 |
|  | Rv-seq | AGGCAGGAGAATCGCTTGAAC | oML #467 |
|  | Fw-seq-Nested | CTTCTTGGAAGAGTGACCTG | oML #468 |
|  | Rv-seq-Nested | CAGTGAGCCAAGATCAAGTC | oML #469 |

**Table S3: Antibodies**

| Antibody | Host | Origin | Western blot | IF |  |
| --- | --- | --- | --- | --- | --- |
| Anti-mouse Alexa 555 | Goat | Thermo fisher Scientific,  A-21424 | - | 1:1000 | aML #015 |
| Anti-mouse IgG CF770 | Goat | Biotum, VWR #20077 | 1:10000 | - | aML #009 |
| Anti-rabbit IgG CF680 | Goat | Biotum, VWR #20067 | 1:10000 | - | aML #010 |
| CHD4 | Rabbit | Active Motif,  39289 | 1:1000 | - | aML #019 |
| CPD | Mouse | Cosmo Bio,  CAC-NM-DND-001 | - | 1:1000 | aML #020 |
| ERCC1 | Mouse | Santa Cruz,  sc-17809 | 1:500 | 1:100 | aML #066 |
| XPF | Mouse | Santa Cruz,  3F2/3, sc-136153 | 1:200 | 1:100 | aML #096 |

**Table S4: *ERCC1* variant properties**

| *ERCC1* Variant  GRCh38 NC_000019.10 | g.45419157G>A | g.45418968-45421119del | g.45414036T>C | g.45419096A>G | g.45413724C>G |
| --- | --- | --- | --- | --- | --- |
| NM_001983.4 | c.466C>T | c.321+61_525+132del | c.703-2A>G | c.525+2T>C | c.796G>C |
| NP_001974.1 | p.Arg156Trp | p? | p? | p? | p.Ala266Pro |
| Exon affected by variant | Exon 4 | Exon 4 and 5 deletion | Exon 8 | Exon 5 | Exon 8 |
| GnomAD frequencies (v4.1.0) | 204 alleles*  0 hom  0.0001276 freq | 0  0 | 13 alleles  0 hom  0.000008067 freq | 0  0 | 5 alleles  1 hom  0.000005125 freq |
| GnomAD frequencies (v4.1.0)  Non UK biobank | 59 alleles  0 hom | 0  0 | 5 alleles  0 hom | 0  0 | 4 alleles  1 hom |
| GnomAD frequencies (v4.1.0)  Heterozygote/homozygote | 204 het */0 hom | 0 het/0 hom | 13 het/0 hom | 0 het/0 hom | 6 hets/1 hom |
| GnomAD frequencies (v4.1.0)  Non UK biobank  Heterozygote/homozygote | 59 het/0 hom | 0 het/0 hom | 5 het/0 hom | 0 het/0 hom | 4 het/1 hom |
| Variant type | Missense | Structural variant  Deletion of 2 exons | Canonical splice variant | Canonical splice variant | Missense |
| SpliceAI | - | - | Splice AI: 0.98 | Splice AI: 0.99 | - |
| REVEL | 0.801 | - | - | - | 0.589 |
| Alpha missense | 0.9557 | - | - | - | 0.976 |
| NMD predicted | No | No - majority of central binding domain absent | Yes | Yes | No |

*Frequency discrepancy between exome and genome allele frequency, noted as statistically significant in gnomAD.  194 alleles were detected in GnomAD exome data compared to 10 alleles in GnomAD genome data, suggesting there may be technical issues with exome data.

Hom: homozygotes

Freq: frequency

**Supplemental references**

1. Apelt K, White SM, Kim HS *et al*: ERCC1 mutations impede DNA damage repair and cause liver and kidney dysfunction in patients. *J Exp Med* 2021; **218**.

2. van der Weegen Y, de Lint K, van den Heuvel D *et al*: ELOF1 is a transcription-coupled DNA repair factor that directs RNA polymerase II ubiquitylation. *Nat Cell Biol* 2021; **23:** 595-607.
